# Vibrotactile auricular vagus nerve stimulation alters limbic system connectivity in humans: A pilot study

**DOI:** 10.1371/journal.pone.0310917

**Published:** 2025-05-29

**Authors:** Kara M. Donovan, Joshua D. Adams, Ki Yun Park, Phillip Demarest, Gansheng Tan, Jon T. Willie, Peter Brunner, Jenna L. Gorlewicz, Eric C. Leuthardt

**Affiliations:** 1 Department of Biomedical Engineering, Washington University, St. Louis, Missouri, United States of America; 2 Division of Neurotechnology, Washington University School of Medicine, St. Louis, Missouri, United States of America; 3 Department of Aerospace and Mechanical Engineering, Saint Louis University, St. Louis, Missouri, United States of America; 4 Department of Neurosurgery, Washington University School of Medicine, St. Louis, Missouri, United States of America; 5 Department of Psychiatry, Washington University School of Medicine, St. Louis, Missouri, United States of America; 6 Department of Neuroscience, Washington University School of Medicine, St. Louis, Missouri, United States of America; 7 Department of Neurology, Washington University School of Medicine, St. Louis, Missouri, United States of America; 8 Department of Mechanical Engineering and Materials Science, Washington University, St. Louis, Missouri, United States of America; 9 Center for Innovation in Neuroscience and Technology, Washington University School of Medicine, St. Louis, Missouri, United States of America; 10 Brain Laser Center, Washington University School of Medicine, St. Louis, Missouri, United States of America; University of Pennsylvania Perelman School of Medicine, UNITED STATES OF AMERICA

## Abstract

Vibration offers a potential alternative modality for transcutaneous auricular vagus nerve stimulation (taVNS). However, mechanisms of action are not well-defined. The goal of this pilot study was to evaluate the potential of vibrotactile stimulation of the outer ear as a method for activating central brain regions similarly to established vagal nerve stimulation methods. Seven patients with intractable epilepsy undergoing stereotactic electroencephalography (sEEG) monitoring participated in the study. Vibrotactile taVNS was administered across five vibration frequencies (2, 6, 12, 20, and 40 Hz) following a randomized stimulation pattern with 30 trials per frequency. Spectral coherence during stimulation was analyzed across theta (4–8 Hz), alpha (8–13 Hz), beta (13–30 Hz), and broadband gamma (70–170 Hz) frequency bands. At the group level, vibrotactile taVNS significantly increased coherence in theta (effect sizes 6 Hz: r = 0.311; 20 Hz: r = 0.316; 40 Hz: r = 0.264) and alpha bands (effect sizes 20 Hz: r = 0.455; 40 Hz: r = 0.402). Anatomically, multiple limbic brain regions exhibited increased coherence during taVNS compared to baseline. The percentage of total electrode pairs demonstrating increased coherence was also quantified at the individual level. Twenty Hz vibration resulted in the highest percentage of responder pairs across low-frequency coherence measures, with a group-average of 33% of electrode pairs responding, though inter-subject variability was present. Overall, vibrotactile taVNS induced significant low-frequency coherence increases involving several limbic system structures. Further, parametric characterization revealed the presence of inter-subject variability in terms of identifying the vibration frequency with the greatest coherence response. These findings encourage continued research into vibrotactile stimulation as an alternative modality for noninvasive vagus nerve stimulation.

## 1 Introduction

Vagus nerve stimulation (VNS) has been widely investigated in both animals and humans for numerous applications [1–11]. To date, surgically implantable VNS has been FDA-approved for intractable epilepsy, treatment-resistant depression, and chronic stroke [12–16]. While VNS has many beneficial effects, its invasive nature inherently limits its applications. Consequently, a noninvasive alternative, transcutaneous auricular VNS (taVNS), is also under investigation for both similar and new indications [17–22]. taVNS targets the afferent auricular branch of the vagus nerve (ABVN), which innervates the cymba concha and tragus regions of the outer ear [23–26]. Importantly, current literature suggests that taVNS activates several of the same regions that are putatively believed to underly the mechanism(s) of action for cervical VNS, namely, the nucleus tractus solitarius (NTS) and locus coeruleus (LC) [24,27–30]. The NTS projects to the LC, through which it has direct and indirect projections to several cortical and subcortical structures, such as the amygdala, hippocampus, and insula, along with other limbic system brain regions [7,24,27].

Given these substantial limbic-relevant connections, invasive and noninvasive VNS have been studied regarding their impact on various cognitive processes, such as memory and attention. Prior imaging literature has demonstrated region-specific responses to VNS in limbic areas with well-documented roles in cognition and memory function, such as the amygdala and cingulate [31–37]. Beyond just activity changes in these areas, VNS has also been shown to enhance theta rhythm synchronization (4–10 Hz) between the basolateral amygdala (BLA) and anterior cingulate cortex (ACC) and increase hippocampal-retrosplenial connectivity in rodent models [38,39].

Current literature suggests that phase synchronization, also referred to as magnitude-squared coherence (MSC), across brain regions plays a role in various cognitive tasks [40–43]. Specifically, noninvasive recordings from human subjects support the notion that there are broad changes in coherence across large cortical regions during memory tasks [44]. Together, these studies suggest that VNS may modulate physiologic mechanisms and cortical and subcortical sites that are relevant to memory and other cognitive functions.

While electrical stimulation is the most common method of noninvasive taVNS, nonelectrical approaches may also activate the ABVN. Addorisio et al. demonstrated that vibrotactile stimulation administered to the cymba concha region of the outer ear could elicit vagal-mediated anti-inflammatory effects by reducing inflammatory markers in healthy adults (two studies: n = 6, decreased endotoxin induced TNF *p* < 0.01; n = 19, decreased TNF *p* < 0.05, IL-1β *p* < 0.001, and IL-6 *p* < 0.01) and lessening inflammatory responses in rheumatoid arthritis (RA) patients (n = 9, decreased DAS-28 score *p* < 0.05) [45]. Additionally, recent work by Tan et al. revealed both arousal and working memory enhancement effects during 6 Hz vibrotactile taVNS (n = 20, increased normalized pupil diameter relative to baseline Cohen’s d = 0.35, *p* = 0.01 and sham Cohen’s d = 0.29, *p* = 0.04, improved *d’* Cohen’s d = 0.63, *p* < 0.01) [46]. While these studies are exciting proofs of concept, what is currently lacking is a more refined understanding of the central effects of vibrotactile taVNS in humans. Thus, we hypothesized that vibrotactile taVNS would induce central brain changes via increased coherence and that these effects would be frequency specific.

Here, we performed a parametric characterization of vibrotactile taVNS, investigating direct cortical and subcortical neurophysiological responses induced by varying vibrotactile taVNS frequencies within invasively-monitored human subjects. Specifically, we quantified coherence changes in several regions of interest (ROIs) – orbitofrontal cortex (OFC), ACC, amygdala, hippocampus, and parahippocampal gyrus (PHG) – using stereotactic electroencephalography (sEEG) recordings from epilepsy patients. ROIs were selected based on brain areas identified from rodent studies and human imaging studies, as well as brain areas that play a role in memory and cognition [6,7,24,28,29,47,48]. This work was designed as a pilot study with the goal of investigating the potential of vibrotactile stimulation as an alternative modality for taVNS. As the focus was on scientific neurophysiological findings rather than clinical, health-related outcomes, this was not a pre-registered clinical trial. Future work will seek to contextualize these neurophysiological findings within a broader clinical framework.

Based on literature indicating that VNS and taVNS have the potential to improve cognitive function, we hypothesized that vibrotactile taVNS would activate a similar pathway and thus increase neuronal communication in brain areas involved in memory [17,49,50]. To test this hypothesis, we identified which brain areas had the greatest response and whether this effect was specific to hypothesized brain regions or rather a whole-brain response. In this study specifically, we used a comprehensive coherence analysis as a means for quantifying neuronal communication. Our results demonstrate that specific frequencies of vibrotactile taVNS induce significant changes in limbic and memory-relevant regions similarly affected by traditional VNS approaches. A larger, pre-registered clinical trial is needed to validate these findings; however, the present work supports the potential of vibrotactile taVNS as a useful noninvasive modality for neuromodulation.

## 2 Materials and methods

### 2.1 Participants

**Fig 1:**
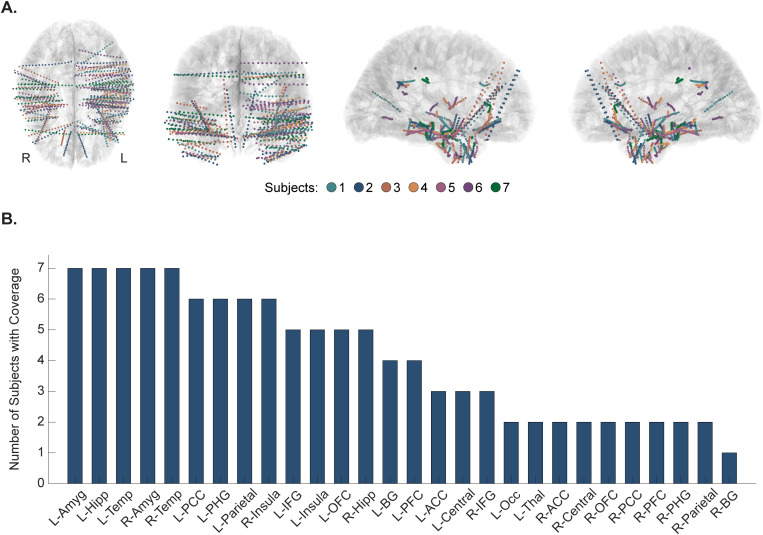
Aggregated anatomical coverage across all subjects. **(A)** Coverage of stereotactic electroencephalography (sEEG) contacts broken down by individual subjects. Each subject’s sEEG coverage is shown in a different color. Images are shown in radiological view. **(B)** Frequency of electrode coverage across anatomical regions. sEEG contacts were classified into 28 different anatomical regions to facilitate group-level analysis. The number of subjects who had coverage in each of these anatomical regions is illustrated in the bar graph (i.e., all 7 subjects had coverage in the left amygdala, left hippocampus, left temporal lobe, right amygdala, and right temporal lobe, while only 1 subject had coverage in the right basal ganglia). ACC = anterior cingulate cortex; Amyg = amygdala; BG = basal ganglia; Hipp = hippocampus; IFG = inferior frontal gyrus; Occ = occipital lobe; OFC = orbitofrontal cortex; PCC = posterior cingulate cortex; PFC = prefrontal cortex; PHG = parahippocampal gyrus; Temp = temporal lobe; Thal = thalamus.

Nine subjects (4 males, 5 females, mean 39 ± 13 years) undergoing clinically-indicated invasive monitoring via sEEG for intractable epilepsy at Barnes Jewish Hospital participated in this study. One subject was excluded due to only having 30 seconds of baseline data and another subject was excluded due to having a large anatomical defect affecting approximately a quarter of the electrodes, resulting in seven subjects for data analysis (2 males, 5 females, mean 36 ± 7 years). Within that population, 16 ± 2 electrode shanks were implanted (200 ± 29 contacts). After adjusting for recording limitations and excluding noisy contacts as well as those classified as white matter, ventricles, or unknown, an average of 174 ± 28 contacts generated good recordings for analysis. Values are reported as mean ± standard deviation unless otherwise stated. Aggregated anatomical coverage spanned 28 brain regions, with predetermined ROIs (OFC, ACC, amygdala, hippocampus, and PHG) exhibiting dense coverage at the population level (Fig 1). The recruitment period for this study took place between May 16, 2022 and April 25, 2023. The study was approved by the Washington University Institutional Review Board prior to enrollment. All subjects were over 18 years of age and provided written informed consent before participating in any research procedures.

### 2.2 Study design

**Fig 2:**
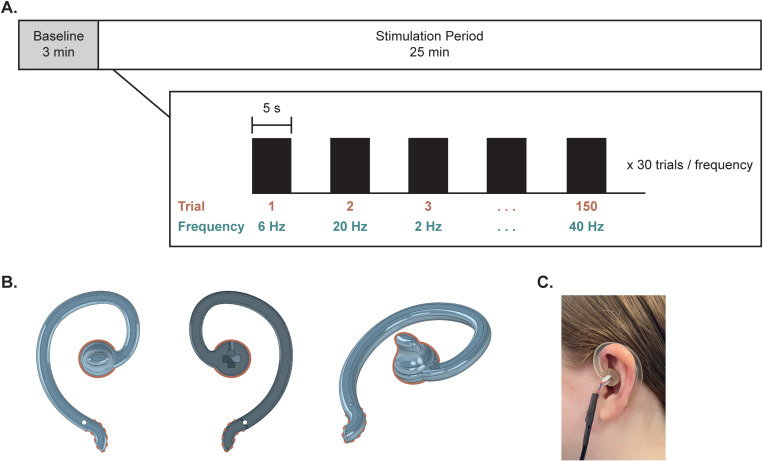
Overall study design and custom earpiece. **(A)** The experimental session consisted of three minutes of baseline followed by a 25-minute stimulation period. During that time, stimulation alternated between five seconds on and five seconds off, with randomized vibration frequencies being delivered. In total, each vibration frequency was delivered for 30 trials, totaling 150 trials across all frequencies. **(B)** A custom, 3D-printed earpiece was used for vibrotactile taVNS, shown from front, back, and angled views. The stimulating end is indicated by a solid line while the stabilizing end is indicated by a dashed line. The stimulating end contacts the cymba concha, and the vibration motor is inserted into the slot visible in the back view. The stabilizing end wraps around the back of the ear for support. **(C)** Image of the device positioned on the ear.

Data collection took place 1–7 days post-implantation and consisted of one experimental session lasting approximately 30 minutes. The session started with three minutes of baseline recorded at rest, followed by five-second stimulation trials alternating with five-second off periods (Fig 2). The off periods were included to allow for a brief washout period between conditions and minimize potential carry-over of effects from one vibration condition to the next. Subjects remained at rest in either their hospital bed or a chair throughout the experimental session and were instructed to stay awake for the protocol. All subjects completed 30 trials for each of five specified vibration frequencies while intracranial activity was recorded continuously. The order of vibration frequencies was randomized, and the subjects did not know the order of upcoming frequencies.

### 2.3 Vibrotactile taVNS

#### 2.3.1 Custom earpiece design.

Because vibration is a new modality for administering taVNS, a custom vibration-delivery earpiece was designed consisting of a flexible hook backbone that wrapped around the top of the ear (Fig 2) [51]. One end (the stimulating end) terminated in the cymba concha, while the other end (the stabilizing end) terminated behind the earlobe. The stimulating end consisted of a friction fit slot through which a 5 mm eccentric rotating mass (ERM) vibration motor was inserted [52]. This design was created in SolidWorks and additively manufactured in Flexible 80A material on a Formlabs Form 3B stereolithographic printer [53]. All printed components were washed in isopropyl alcohol and underwent heated post-cure per Formlabs specification prior to use. Vibration characteristics were also validated via Laser Doppler Vibrometry (LDV) benchtop testing to ensure specified frequencies were being generated.

#### 2.3.2 Administration of taVNS.

Vibrotactile taVNS was administered across five pulsed frequencies – 2, 6, 12, 20, and 40 Hz – selected to span physiologically relevant frequency bands. These vibration frequencies were specifically chosen under the hypothesis that one of these rhythms would best resonate with underlying neural circuitry. Based on literature suggesting neuronal responses to VNS and taVNS are temporally precise, a five-second trial duration was used [54–58]. Stimulation alternated between five seconds on, five seconds off, with each vibrotactile condition delivered for 30 trials. Trials were randomly interleaved for a total of 150 vibrotactile trials across all frequencies.

### 2.4 Data acquisition

sEEG signals were recorded at a 2,000 Hz sampling rate using the general-purpose BCI2000 software package [59]. A Nihon Kohden JE-120 A recording system (Nihon Kohden, Tokyo, Japan) was used to amplify and digitize the sEEG signals.

### 2.5 Data analysis

#### 2.5.1 Electrode localization.

Localization of sEEG electrodes was performed using the Versatile Electrode Localization Framework (VERA) [60]. The postoperative computed tomography (CT) scan was first registered to the preoperative magnetic resonance imaging (MRI) scan to generate a subject-specific brain model. Electrodes were then mapped to anatomical labels based on the aparc + aseg parcellation scheme using the Desikan-Killiany atlas. To reduce dimensionality and facilitate group analysis, these FreeSurfer labels were grouped together structurally into 28 brain regions (S1 Table). For example, electrodes labeled as left rostral anterior cingulate and left caudal anterior cingulate were grouped into one region (left anterior cingulate cortex, L-ACC) and electrodes labeled as left medial orbitofrontal and left lateral orbitofrontal were grouped into one region (left orbitofrontal cortex, L-OFC). For visualization purposes, electrode coverage was plotted on the MNI152 atlas in volume space (S1 Fig). For example, responses in the L-ACC region were visualized by shading the FreeSurfer-defined parcels for left rostral and caudal anterior cingulate because electrode coverage spanned both those regions.

#### 2.5.2 sEEG preprocessing.

Preprocessing was performed via custom scripts implemented in MATLAB R2021a (https://github.com/neurotechcenter/vibrotactile_taVNS_coherence). DC offset was first removed from raw sEEG signals using a fourth-order Butterworth highpass filter with a cutoff frequency at 0.5 Hz. Then, channels with excessive 60 Hz line noise, defined as having 60 Hz power exceeding three mean absolute deviations above the median, were identified and excluded from future analysis. Data was re-referenced using common average referencing (CAR) before applying a 60 Hz notch filter to remove line noise.

Baseline data was then extracted and broken into five-second epochs to match the duration for vibrotactile stimulation trials, facilitating a baseline subtraction normalization approach. Stimulation data was also broken into epochs specific to each vibration frequency. To minimize effects of interictal epileptic activity, trials with a spike exceeding 500 µV (absolute value) were excluded from future analysis (S2 and S3 Figs).

#### 2.5.3 Spectral coherence analysis.

All coherence analyses were also conducted via custom scripts implemented in MATLAB R2021a (https://github.com/neurotechcenter/vibrotactile_taVNS_coherence). Spectral coherence was computed across four canonical frequency bands – theta (4–8 Hz), alpha (8–13 Hz), beta (13–30 Hz), and broadband gamma (70–170). To expedite processing time during coherence calculations, the data was downsampled to 500 Hz. Coherence was computed using the magnitude-squared coherence function in MATLAB (mscohere) with a window size of 2.5 seconds and 70% overlap.


$$\mathrm{\backslash [}C_{xy}(f)=\;\frac{{\left|P_{xy}(f)\right|}^{2}}{P_{xx}(f)P_{yy}(f)}\cdot \mathrm{\backslash ]}$$

*P*_*xy*_ represents the cross power spectral density (PSD) of *x* and *y*, and *P*_*xx*_ and *P*_*yy*_ represent the PSDs of *x* and *y*, respectively. To normalize the data and facilitate group-level analyses, we performed a baseline subtraction on all coherence values.

#### 2.5.4 Signal-to-noise ratio.

To identify the vibration frequency/frequencies that elicited the most consistent coherence increase across subjects, we defined a signal-to-noise ratio (SNR), SNR=μ/σ. The SNR was computed for each of the five vibration frequencies by calculating the mean, µ, and standard deviation, *σ*, of the responder percentages from all subjects for the given vibration frequency.

### 2.6 Statistical analysis

Nonparametric statistical analyses (due to non-normally distributed data) were performed in MATLAB R2021a and RStudio, with RStudio being used to run Friedman tests and the associated post-hoc tests as well as the exact multinomial test. At the group level, Friedman tests were used as a nonparametric alternative to a repeated measures analysis of variance to compare group average coherence changes between vibration conditions. When indicated by a significant result, post-hoc two-tailed pairwise Wilcoxon signed-rank tests (Bonferroni-corrected) were used to identify which vibration frequencies were significantly different from each other, and the effect size was computed as $r=Z/\sqrt{}N$, where *Z* represents the *Z*-statistic and *N* represents the sample size. To test whether vibrotactile taVNS altered coherence across relevant brain regions relative to baseline, two-tailed, one-sample Wilcoxon signed-rank tests were also used to identify distributions of MSC changes significantly different than 0. The significance threshold was corrected for multiple comparisons (Bonferroni-corrected, 5 vibration conditions tested). Responder pairs across each vibration frequency for individual subjects were identified as those with a Cohen’s d greater than 0.2 when comparing stimulation to baseline. This threshold for identifying a small effect was chosen based on the prevalence of inter-subject variability in electrophysiology studies [61–64]. Additionally, Kruskal-Wallis tests were also used at the group level for comparing unequal distributions of threshold-based coherence changes in the ROI-driven analysis.

Friedman tests were also used at the individual subject level to compare MSC during vibration to baseline across all five vibration frequencies. When indicated by a significant result, post-hoc two-tailed Wilcoxon signed-rank tests (Bonferroni-corrected) were again used to identify the significant frequency condition(s). This allowed for identification of which frequencies resulted in the strongest global coherence responses for each subject. The exact multinomial test was used to quantify whether the rankings of vibration frequencies were statistically significant.

Finally, permutation testing was used to assess SNR results by quantifying whether the observed SNR values were significantly greater than what would be expected by chance. 10,000 permutations were used, shuffling the vibration condition labels (2, 6, 12, 20, and 40 Hz) for each subject and computing the resulting SNR values. For each condition, *p*-values were then computed as the proportion of iterations where the permuted SNR was greater than the observed SNR.

## 3 Results

### 3.1 Global coherence analysis

To assess for brain-wide coherence changes during vibrotactile taVNS, the group-average coherence change from baseline across all possible brain region pairs was aggregated together into a global distribution (n = 353 pairs). To assess differences between vibration frequencies, a Friedman test (data non-normally distributed, Shapiro-Wilk test all *p* < 0.05) was first conducted, revealing significant differences between at least two conditions for all four physiological frequency bands (theta: χ^2^_(4)_ = 140.88, *p* = 1.83 x 10^-29^; alpha: χ^2^_(4)_ = 150.04, *p* = 2.00 x 10^-31^; beta: χ^2^_(4)_ = 61.63, *p* = 1.32 x 10^-12^; gamma: χ^2^_(4)_ = 51.13, *p* = 2.09 x 10^-10^). Post-hoc two-tailed Wilcoxon signed-rank tests (Bonferroni-corrected) identified specific pairs of vibration conditions that were significantly different (effect sizes and *p*-values for all pairwise tests found in S2 Table). Theta coherence was significantly different between all vibration condition pairs except for 6, 20, and 40 Hz, which were not different from each other. Alpha coherence was also different between all vibration pairs except 6 and 12 Hz and 20 and 40 Hz. Beta coherence had less of an effect, with differences during 2 Hz vibration compared to 6, 20, and 40 Hz, as well as during 12 Hz vibration compared to 6 and 40 Hz. Similarly, gamma coherence was less affected, with differences during 2 Hz vibration compared to 6, 20, and 40 Hz, as well as during 6 Hz compared to 12, 20, and 40 Hz.

**Fig 3:**
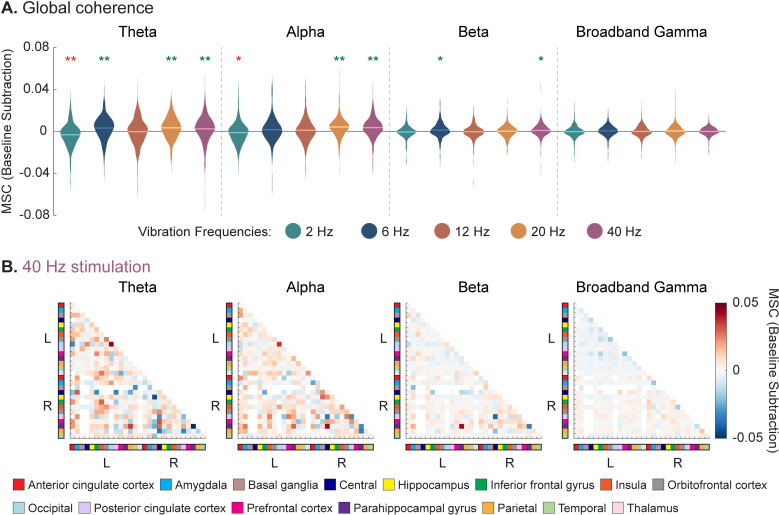
Group-level distributions of magnitude-squared coherence (MSC) changes across theta, alpha, beta, and broadband gamma physiological frequency bands. **(A)** MSC changes relative to baseline are compared for five vibration frequencies – 2, 6, 12, 20, and 40 Hz. The distributions shown encompass coherence changes for all possible pairs of anatomical regions, with the solid line representing the global mean (i.e., average coherence change across all pairs). Asterisks depict differences from zero (green asterisks indicate an increase, red asterisks indicate a decrease), while pairwise differences can be found in S2 Table. Significance threshold Bonferroni-corrected for multiple comparisons. Two-tailed, one-sample Wilcoxon signed-rank test: * = Bonferroni-corrected *p* < 0.05; ** = Bonferroni-corrected *p* < 0.001. **(B)** For a given vibration frequency, MSC matrices are shown for each physiological frequency band to delineate which brain regions exhibit notable coherence increases. Voxels that are completely white indicate that no subjects had coverage spanning those brain region pairs (e.g., no subjects had coverage in both right OFC and left central cortex).

To then assess whether global coherence significantly differed from baseline for each vibration condition, two-tailed, one-sample Wilcoxon signed-rank tests (Bonferroni-corrected) were performed. Vibrotactile taVNS significantly increased MSC in the theta band as compared to baseline when administered at vibration frequencies of 6, 20, and 40 Hz (6 Hz: r = 0.311, *p* = 2.46 x 10^-8^; 20 Hz: r = 0.316, *p* = 1.48 x 10^-8^; 40 Hz: r = 0.264, *p* = 3.64 x 10^-6^) and decreased theta MSC at 2 Hz (r = 0.299, *p* = 9.50 x 10^-8^) (Fig 3). Alpha coherence was also significantly increased during 20 and 40 Hz vibration (20 Hz: r = 0.455, *p* = 5.75 x 10^-17^; 40 Hz: r = 0.402, *p* = 2.22 x 10^-13^) and decreased during 2 Hz vibration (r = 0.169, *p* = 0.008). Beta coherence exhibited marginal increases during 6 and 40 Hz (6 Hz: r = 0.153, *p* = 0.021; 40 Hz: r = 0.178, *p* = 0.004). No significant changes were observed for broadband gamma coherence (all *p* > 0.05). All *p*-values are reported as adjusted for multiple comparisons. Based on these results suggesting the most significant coherence effects were occurring in the lower frequency bands (i.e., theta and alpha), subsequent analyses focused on these frequencies to better resolve key brain regions responding to the stimulation.

### 3.2 Subject-specific frequency analysis

**Fig 4:**
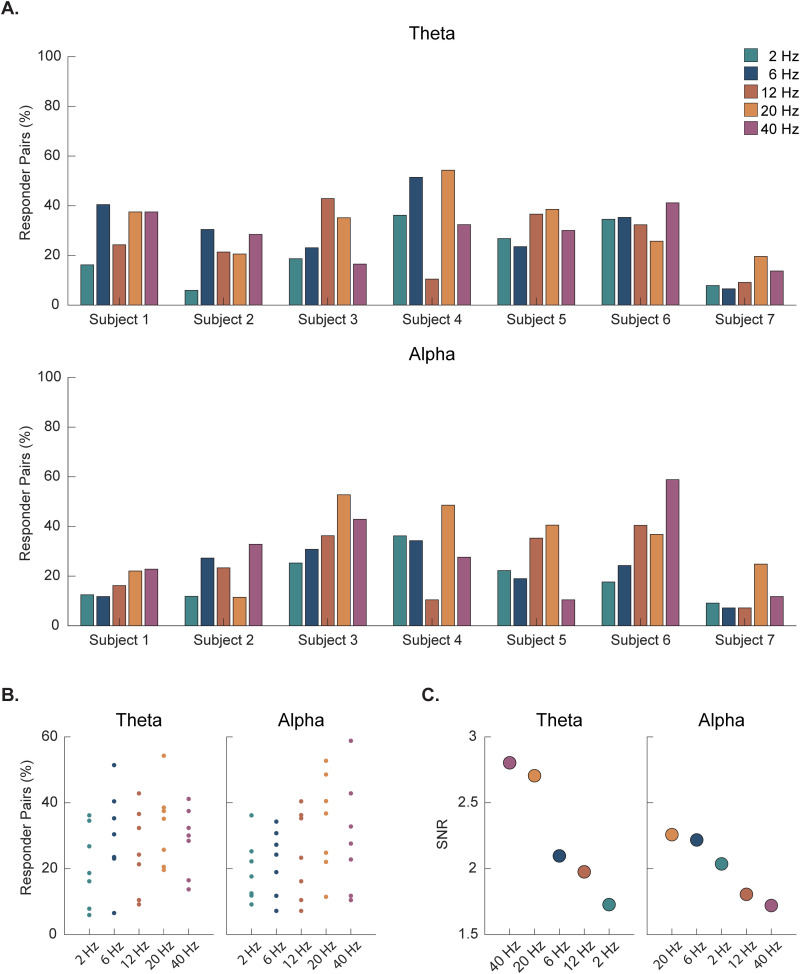
Responder pairs for individual subjects in the theta and alpha frequency bands for all vibration conditions. **(A)** Responders are defined as pairs of electrodes with a Cohen’s d greater than 0.2 when comparing the mean response during stimulation vs. baseline. Responder pairs are shown as a percentage of total pairs. Subject specificity is present as subjects have unique vibration conditions that elicit the greatest percent of responses. **(B)** Data from **(A)** is also represented by vibration frequency to illustrate variability across subjects in terms of the percentage of responder pairs. **(C)** A signal-to-noise ratio (SNR) was computed for each vibration frequency based on the individual subject measurements to generate an overall ranking at the group level.

To assess which vibration frequency condition(s) subjects were responding to most strongly, we established a responder criterion. Within a subject-specific distribution of all possible electrode pairs, a pair was classified as a responder if the Cohen’s d for stimulation compared to baseline was greater than 0.2. To facilitate comparisons across subjects, we then converted the total number of responder pairs per subject to a percentage of total pairs. Our findings demonstrate that there is subject specificity in terms of which vibration frequency resulted in the maximal coherence response (Fig 4). Regarding theta coherence, two subjects had the highest responder percentage to 6 Hz vibration, one subject to 12 Hz vibration, three subjects to 20 Hz vibration, and one subject to 40 Hz vibration (Fig 4A). An exact multinomial test indicated these rankings are not statistically significant (*p* = 0.624). For alpha coherence, subject variability was less, with four subjects exhibiting the highest responder percentage to 20 Hz vibration and three subjects to 40 Hz vibration (Fig 4A). These rankings are significantly different from random (exact multinomial test, *p* = 0.016). We also computed SNR (**Section** **3.5.4**) for each vibration frequency and then ranked them in descending order to quantify response consistency. The top two frequencies that elicited the most consistent theta coherence increase were 40 and 20 Hz, while 20 and 6 Hz elicited the most consistent alpha coherence increase. However, permutation testing revealed that the SNR values were not significantly greater than random chance for both theta (2 Hz *p* = 0.90, 6 Hz *p* = 0.59, 12 Hz *p* = 0.71, 20 Hz *p* = 0.18, and 40 Hz *p* = 0.15) and alpha coherence (2 Hz *p* = 0.39, 6 Hz *p* = 0.25, 12 Hz *p* = 0.61, 20 Hz *p* = 0.23, and 40 Hz *p* = 0.69).

### 3.3 Exemplar subject

**Fig 5:**
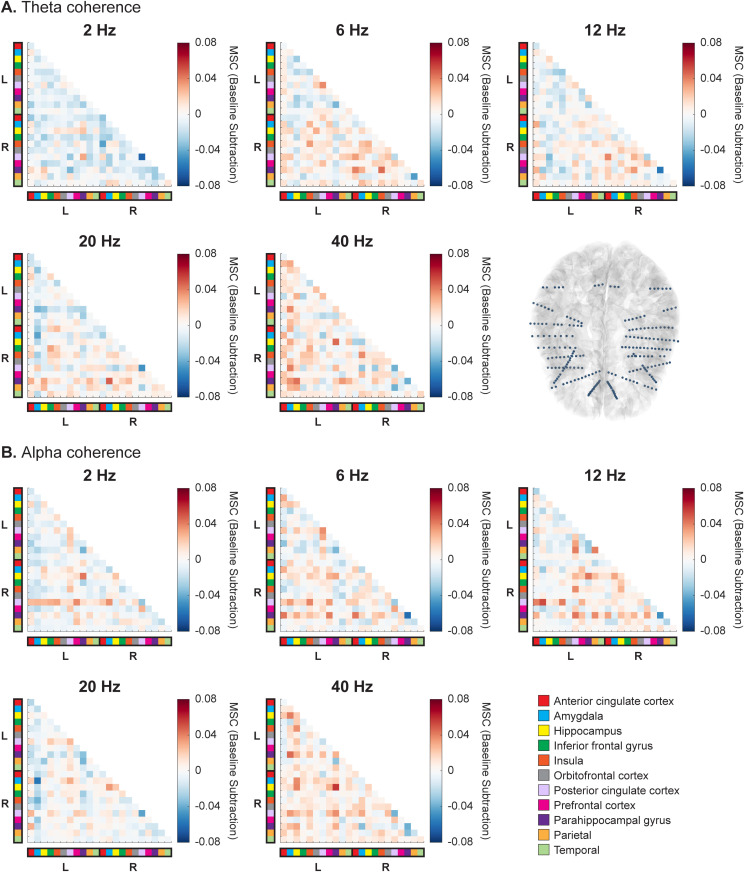
Exemplar subject magnitude-squared coherence (MSC) in the theta (A) and alpha (B) frequency bands for all anatomical regions. Coherence matrices are shown for all five vibration frequencies, where each square voxel represents the average coherence during stimulation (relative to baseline) for a given pair of brain regions. The color blocks along the axes correspond to a particular brain region (bottom right of **B**) and are grouped by left and right hemisphere. The selected exemplar subject had comparable bilateral electrode coverage across both hemispheres, spanning 11 brain regions per hemisphere for a total of 22 regions (as depicted in the bottom right of **A**).

Due to the demonstrated subject specificity (Fig 4), we also analyzed individual subjects at the electrode level. Region-based average coherence matrices for an exemplar subject are shown, with the selected subject chosen based on bilateral coverage of electrode placement overlapping with all five predetermined ROIs (Fig 5, complete coherence matrices for all electrodes in S4 Fig). As demonstrated in the coherence maps, this subject had strong coherence increases in both theta and alpha during 40 Hz vibration, as well as a theta increase during 6 Hz vibration (Fig 5). A Friedman test (data non-normally distributed, Shapiro-Wilk test all *p* < 0.05) was implemented to compare the region-specific coherence values across all five vibration frequencies against the baseline, revealing significant differences between the conditions (theta: χ^2^_(5)_ = 219.16, *p* = 2.24 x 10^-45^; alpha: χ^2^_(5)_ = 149.32, *p* = 1.86 x 10^-30^). Post-hoc two-tailed Wilcoxon signed-rank tests (Bonferroni-corrected) were then conducted to determine which vibration frequencies significantly altered coherence (S5 Fig). Theta coherence was significantly increased during 6 and 40 Hz vibration and significantly decreased during 2 Hz vibration (all *p* < 0.001). Specific to alpha coherence, results indicated a significant increase during 40 Hz vibration and a significant decrease during 2 and 20 Hz vibration (all *p* < 0.001).

### 3.4 Data-driven responders

**Fig 6:**
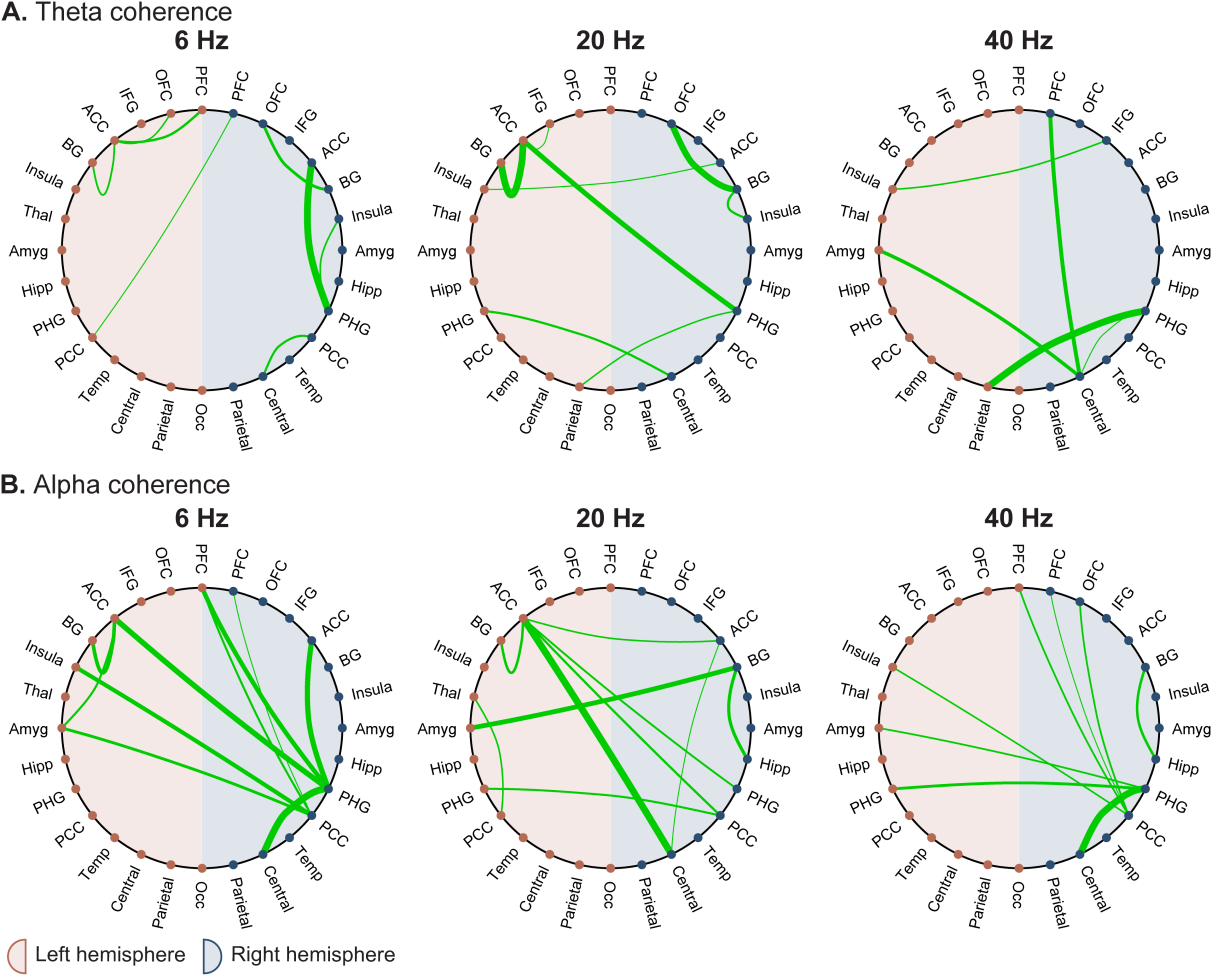
Theta (A) and alpha (B) coherence pairs that are at least 2 standard deviations above the mean for 6, 20, and 40 Hz vibration conditions as identified in Fig 3. Only coherence pairs that are ≥ 2 standard deviations above the mean are shown, and the line thickness is relative to the magnitude, such that the thickest line in each plot is the pair with the greatest coherence increase. Note that the line thickness is relative to the particular plot (i.e., each pairing with the thickest line does not have the same magnitude coherence increase; rather, each pairing with the thickest line represents the greatest coherence increase shown in that plot). All coherence pairings can be found in S6 Fig. Left regions are denoted with red points while right regions are denoted with blue points. Brain regions are sorted from anterior to posterior when looking from top to bottom of the left or right hemisphere. ACC = anterior cingulate cortex; Amyg = amygdala; BG = basal ganglia; Hipp = hippocampus; IFG = inferior frontal gyrus; Occ = occipital lobe; OFC = orbitofrontal cortex; PCC = posterior cingulate cortex; PFC = prefrontal cortex; PHG = parahippocampal gyrus; Temp = temporal lobe; Thal = thalamus.

We then took a data-driven approach to identify which electrode pairs exhibited the strongest coherence changes during vibration. As demonstrated in Fig 3, vibration frequencies of 6, 20, and 40 Hz resulted in statistically significant global coherence increases in the theta and alpha bands. Electrode pairs with a coherence increase of ≥ 2 standard deviations above the brain-wide mean were identified (Fig 6). These pairings correspond to the points in the top portion of the brain-wide distributions above (Fig 3). Brain regions with significant coherence increases to two or more brain regions were left ACC (theta – 6, 20 Hz; alpha – 6, 20 Hz), left PFC (alpha – 6 Hz), left amygdala (alpha – 6 Hz), right ACC (alpha – 20 Hz), right BG (theta – 20 Hz; alpha – 20 Hz), right PHG (theta – 6, 20, 40 Hz; alpha – 6, 40 Hz), right posterior cingulate cortex (PCC) (alpha – 6, 20, 40 Hz), and right central cortex (theta – 40 Hz; alpha – 20 Hz). Complete group-average connectivity matrices can be found in S6 Fig.

### 3.5 Region- and frequency-specific responses

**Fig 7:**
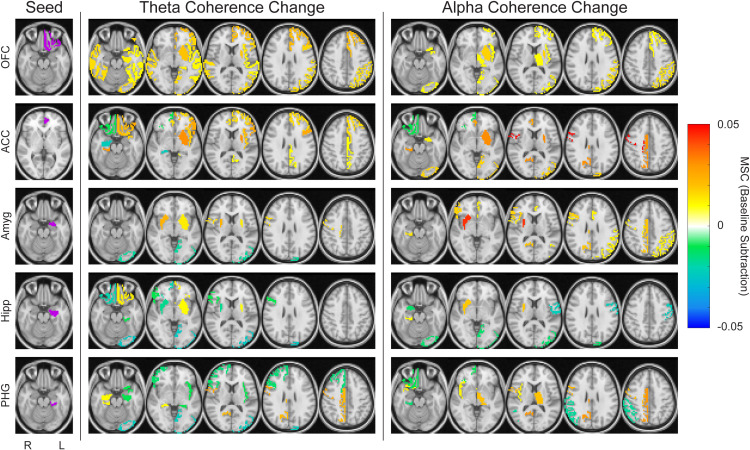
Coherence changes to identified regions of interest (ROIs) at the group level. ROIs were set as the seed region, and coherence changes are shown to the rest of the regions encompassed in the sEEG coverage. A threshold of 0.01 was set such that only changes with an absolute value > 0.01 are shown. Both theta and alpha coherence changes are shown, all under the 20 Hz vibration condition. Increased coherence during vibration is shown as a positive coherence change (warm tones), while decreased coherence during vibration is shown as a negative coherence change (cool tones). From top to bottom, seeds are left orbitofrontal cortex (OFC), left anterior cingulate cortex (ACC), left amygdala (Amyg), left hippocampus (Hipp), and left parahippocampal gyrus (PHG). Images are shown in radiological view. The highlighted seed regions are mapped based on predetermined groups of FreeSurfer parcellations to reduce dimensionality (see S1 Table and S1 Fig).

Predetermined ROIs were defined as seeds to test the hypothesis that vibrotactile taVNS would result in increased coherence along the vagal activation pathway. Changes in theta and alpha coherence during vibrotactile stimulation compared to baseline occurred in anatomically distinct brain regions (Fig 7). Specifically, each anatomic seed (left OFC, left ACC, left amygdala, left hippocampus, and left PHG) had distinct coherence change maps involving a varied set of brain regions. Coherence changes were dependent not only on the anatomic seed, but also on the physiological frequency band.

Across the seeds, there was variability in terms of both magnitude and direction of theta and alpha coherence changes. Specifically, during 20 Hz stimulation and with the OFC as a seed, there was overlap in directional changes in theta and alpha coherence with five brain regions exhibiting increased theta and alpha coherence with the OFC. The amygdala, on the other hand, exhibited more distinct theta and alpha coherence change maps, with only two brain regions having increased theta and alpha coherence. See S3 Table for the complete breakdown of all seeds and all vibration frequencies.

**Fig 8:**
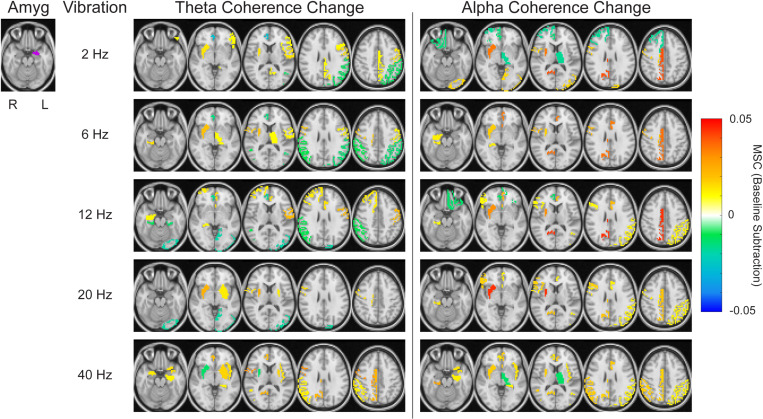
Theta and alpha coherence changes across all five vibration conditions for a specific seed region of interest. The left amygdala was chosen as the seed due to all subjects having electrode coverage in that region. As in Fig 7, a threshold of 0.01 was set, and increased coherence during vibration is shown as a positive coherence change, while decreased coherence during vibration is shown as a negative coherence change. Images are shown in radiological view.

Conversely, to isolate effects specific to the vibration frequency parameter, the anatomic seed was held constant while comparing coherence patterns. This analysis similarly resulted in diverse anatomic patterns of theta and alpha coherence dependent on the vibration frequency condition (Fig 8, see also S7 and S8 Figs and S3 Table). Also notable is that the different vibration conditions elicited different anatomic distributions of coherence changes. Kruskal-Wallis tests indicated significant differences in the threshold-applied theta and alpha coherence maps across vibration conditions with both the OFC and ACC as seeds (OFC theta coherence *p* = 0.024, alpha coherence *p* = 0.039; and ACC theta coherence *p* = 0.033, alpha coherence *p* = 0.001). No significant differences were present with the amygdala, hippocampus, or PHG as seeds (all *p* > 0.05; see S4 Table for complete *p*-values across all seeds). Pairwise comparisons for seeds with significant results can be found in S5 Table.

## 4 Discussion

We investigated the potential of vibrotactile stimulation administered to the cymba concha region of the outer ear as an alternative modality for taVNS by investigating resulting neuronal coherence. To do so, we characterized neurological responses to vibration at varying frequencies in subjects undergoing continuous sEEG monitoring. The present work demonstrates that vibrotactile taVNS activates key brain regions purported to underly mechanism(s) of action for VNS and taVNS. Specifically, vibrotactile taVNS at 6, 20, and 40 Hz increased global, low-frequency coherence. For most subjects, at least 20% of electrode pairs exhibited a Cohen’s d of > 0.2 when comparing coherence during those vibration conditions to baseline. When thresholding for pairs with a coherence increase of at least two standard deviations above the mean, brain regions emerging as hubs with increased low-frequency coherence to multiple areas included the left ACC, PFC, and amygdala and the right ACC, PHG, BG, PCC, and central cortex.

### 4.1 Group-level characterization of vibrotactile taVNS in humans

At the group level, our results suggest an overall increase in global low-frequency (theta and alpha) coherence, most evident during 6, 20, and 40 Hz vibration (Fig 3). Of note, the effect sizes for global coherence increase across these vibration conditions were relatively small (range of r = 0.264–0.455). This finding has several possible interpretations, which will require further research to discern. One possibility is that our experimental design of five seconds on, five seconds off provided too short a stimulation duration to achieve maximum coherence effects, and longer periods might yield larger effects. Another possible explanation is that the stimulation intensity was insufficient to maximally activate the ABVN mechanically. Alternatively, vibrotactile taVNS may inherently result in small yet significant changes in low-frequency coherence, with more intense or prolonged stimulation unlikely to increase the effect size. This is plausible given that studies on intracranial responses to both traditional VNS and electrical taVNS have reported meaningful electrophysiological responses with small magnitude effect sizes [64,65]. To elucidate which of these most accurately reflects the mechanism of vibrotactile taVNS, a larger, multi-faceted study is needed.

Studies have shown that tasks requiring enhanced arousal and higher cognitive load, such as spatial conflict or working memory tasks, are associated with increases in theta and alpha coherence [66–69]. Of note, central vagus nerve projections, such as those activated by VNS and taVNS, influence arousal, so it is possible that this increased arousal is contributing to the increase in coherence [70–74]. While the mechanisms for our observed parametric specificity in terms of stimulation frequency are not clearly understood, we hypothesize that the rhythm structure of the sensory input may have different resonances with underlying neural circuitry. Moreover, insights can be drawn from other neuromodulatory approaches. For example, it has been shown that repetitive transcranial magnetic stimulation (rTMS) can increase alpha coherence involving the ipsilateral motor area, as well as increase directed alpha coherence from frontal to parietal lobes [75,76]. While not directly related to the present study on vibrotactile taVNS, these rTMS studies both observed increased alpha coherence while administering stimulation at unique frequencies (1 Hz and 10 Hz, respectively).

The specificity of responses depending on vibration frequency complements current literature on electrical VNS and taVNS studies, which have demonstrated frequency-specific effects [54,77–80]. Additionally, the anatomic locations of cortical and subcortical regions that demonstrated the most robust coherence changes are structurally distinct. Thus, it is consistent that frequency rhythms known to be associated with longer-range interactions (i.e., theta, alpha) would demonstrate preferential effects [81,82].

To anatomically resolve the regions that demonstrated enhanced coherence, we performed a data-driven analysis to classify sites with the most robust responses to stimulation (Fig 6). Overlapping with our initial hypothesis, the ACC and PHG were key regions that emerged as hubs. These findings align with rodent models by Cao et al. (2016) that revealed an increased theta band correlation between the basolateral amygdala and ACC following VNS [38].

We further analyzed brain-wide responses within theta and alpha coherence in predefined cortical and subcortical ROIs. Several limbic ROIs exhibited increased coherence to each other, and the thalamus and PCC emerged as other sites with increased coherence to multiple ROIs (Fig 7). The limbic system and thalamus have been well-studied in the context of VNS [37,83,84]. Our results demonstrate that they have enhanced connectivity, which supports the notion that vibrotactile taVNS activates central structures similar to classic electrical VNS/taVNS. Of note, the coherence effects across these ROIs varied depending on whether theta or alpha rhythms were assessed. ROI coherence was also dependent on vibration frequency (Fig 8). Parametric analyses of VNS have shown the importance of parameter selection in terms of both physiological and behavioral responses, and our work is consistent in illustrating that vibration frequency selection is also relevant in the context of vibrotactile taVNS. A multitude of factors may explain this parametric sensitivity of neuronal responses. The diverse projections stemming from the vagus nerve and the variable proximity of other nuclei to the vagus nerve termination site may lead to distinct temporal sensitivities [54].

### 4.2 Subject-specific frequency responses to vibrotactile taVNS

Individualized responses to neuromodulation techniques and broader instances of personalized medicine are well-documented in the literature [85–90]. Accordingly, we also assessed variance in response to the different vibrotactile parameters across individuals. For the purposes of this study, we defined a subject’s most effective frequency as that which resulted in the greatest percentage of electrode pairs classified as responders. While the same three frequencies emerged through this analysis as at the group level (6, 20, and 40 Hz), inter-subject variability was present in terms of which of those frequencies resulted in the most widespread coherence increases (Fig 4). As an example, we highlighted the MSC changes in Subject 2, who had near-symmetrical bilateral coverage (Fig 5). However, it is important to note that inter-subject response variability may be somewhat attributable to the inter-subject variability in electrode coverage (Fig 1). Additionally, some variability in baseline coherence was present amongst participants, which could also influence our results (S9 Fig and S6 Table). This variability in baseline brain states was expected, though, so to account for this, we normalized data via baseline subtraction prior to aggregating individual data at the group level. Further, despite the individual response variation across vibration frequencies, there were vibration parameters that elicited more consistent central responses. Specifically, 20 Hz vibration frequency had a high SNR, albeit not statistically significant, for inducing both theta and alpha coherence increases. For theta specifically, however, 40 Hz stimulation had a slightly higher SNR. Moving forward, we propose future studies investigate 20 Hz vibrotactile taVNS as it maximized the SNR across theta and alpha in our cohort and had the highest effect size for increased global coherence in those bands. Alternatively, to customize vibrotactile taVNS and achieve maximum coherence increases in an individual patient, further studies are needed to identify and validate noninvasive means for selecting the most effective stimulation parameters for a particular patient.

### 4.3 Limitations

To collect valuable invasive brain recordings from human subjects, data was recorded from patients with intractable epilepsy undergoing invasive monitoring via sEEG. These recordings offer critical insights into how cortical and subcortical structures such as the amygdala and hippocampus respond to vibrotactile taVNS. However, we note that data from a clinical cohort may not be indicative of wider populations and that an sEEG cohort presents added constraints regarding data collection. While recordings were conducted in a naturalistic environment without a fixed eyes open or closed state, individual subject data were normalized via baseline subtraction from the same session. Future studies with other clinical populations or healthy controls using noninvasive neuroimaging modalities (e.g., fMRI, EEG) may enhance the reproducibility and translatability of our present findings by providing opportunities to control for time of day of stimulation as well as to compare across different environments. Further, the study is limited by the sample size; however, given the novelty of vibrotactile taVNS, the goal was to determine whether there is a central response to justify continued research. To our knowledge, there have only been two prior studies that investigated the use of vibration as an alternative taVNS modality. The first study sought to extend known anti-inflammatory effects of invasive VNS to a vibrotactile modality, revealing beneficial anti-inflammatory effects [45,91,92]. Another study demonstrated the potential of 6 Hz vibration administered to the cymba concha to improve working memory performance, while also highlighting the ability of vibrotactile taVNS to increase general arousal during the course of repeated working memory tasks [46]. While direct comparisons cannot be drawn between these studies and this work, they provided valuable evidence supporting the use of vibration as an alternative to electrical stimulation to activate the ABVN. Through this pilot study, we aimed to better understand underlying mechanisms to support continued investigation of this modality. An additional limitation of this work concerns the sound produced by vibration motors. While administering vibrotactile stimulation to the outer ear, the stimulation caused both mechanical and auditory perception. Future work should investigate whether auditory stimulation alone without mechanosensation results in similar coherence changes in the brain.

## 5 Conclusions

Vibrotactile taVNS offers an alternative approach to activating vagal fibers to influence central brain dynamics. The present work leveraged human intracranial recordings to demonstrate the viability of vibrotactile taVNS to enhance coherence between central cortical and subcortical structures known to be associated with classic electrical VNS stimulation. Further, the central effects are subject-specific and can be variable depending on both the frequency band assessed and the frequency of vibrotactile stimulation.

## Supporting information

S1 Table Aggregated FreeSurfer groups. FreeSurfer parcellations were grouped structurally into 28 defined brain regions to reduce the dimensionality. ACC = anterior cingulate cortex; Amyg = amygdala; BG = basal ganglia; Hipp = hippocampus; IFG = inferior frontal gyrus; OFC = orbitofrontal cortex; Occ = occipital lobe; PCC = posterior cingulate cortex; PFC = prefrontal cortex; PHG = parahippocampal gyrus; Temp = temporal lobe; Thal = thalamus.
(DOCX)

S2 Table Pairwise comparisons of global coherence changes. Bonferroni-corrected post-hoc pairwise tests were run based on statistically significant differences in the Friedman test. Results are reported for all four physiological frequency bands, with significant pairs indicated (corrected *p*-values). * *p* < 0.05; ** *p* < 0.01.
(DOCX)

S3 Table Threshold-applied responses to ROIs. For each of five ROIs, brain regions with a coherence change of at least 0.01 (absolute value) are listed. Response regions are categorized by both vibration frequency (2, 6, 12, 20, and 40 Hz) and coherence frequency (theta and alpha). The ROIs were set as the seed and the coherence was computed with all other brain regions, and then the response threshold was applied. The regions listed here correspond to the regions depicted visually in Fig 8 and S7 and S8 Figs. Note that the regions listed here are based on the response threshold rather than a statistical test. ACC = anterior cingulate cortex; Amyg = amygdala; BG = basal ganglia; Hipp = hippocampus; IFG = inferior frontal gyrus; OFC = orbitofrontal cortex; Occ = occipital lobe; PCC = posterior cingulate cortex; PFC = prefrontal cortex; PHG = parahippocampal gyrus; Temp = temporal lobe; Thal = thalamus.
(DOCX)

S4 Table Comparisons of group-level coherence responses to ROIs. Kruskal-Wallis tests were run to compare the threshold-applied response distributions of theta and alpha coherence changes to each of five ROIs. The ROIs were set as seeds and coherence values to all other brain regions were computed. A response threshold was set at 0.01 (absolute value). All seeds are in the left hemisphere. * *p* < 0.05; ** *p* < 0.01.
(DOCX)

S5 Table Pairwise comparisons of group-level coherence responses to significant ROIs. Bonferroni-corrected post-hoc pairwise tests were run based on statistically significant differences in the Kruskal-Wallis test (S4 Table). Results are reported for both theta and alpha coherence distributions, with significant pairs indicated (corrected *p*-values). * *p* < 0.05; ** *p* < 0.01.
(DOCX)

S6 Table Pairwise comparisons of baseline coherence. Bonferroni-corrected post-hoc pairwise tests were run based on statistically significant differences in the Kruskal-Wallis test. Results are reported for all four physiological frequency bands, with significant pairs indicated (corrected *p*-values). * *p* < 0.05; ** *p* < 0.01.
(DOCX)

S1 Fig All FreeSurfer parcellations included in anatomical analysis. ACC = anterior cingulate cortex; Amyg = amygdala; BG = basal ganglia; Hipp = hippocampus; IFG = inferior frontal gyrus; Occ = occipital lobe; OFC = orbitofrontal cortex; PCC = posterior cingulate cortex; PFC = prefrontal cortex; PHG = parahippocampal gyrus; Temp = temporal lobe; Thal = thalamus.
(PDF)

S2 Fig Rejecting trials with interictal epileptiform discharges. **(A)** All preprocessed baseline epochs (5 s) from one electrode in one subject. Any trial with a positive or negative spike exceeding abs(500) µV (two trials highlighted here in teal and pink) was marked as containing epileptiform discharges and excluded from future analysis. **(B)** This example electrode is located in the right amygdala.
(PDF)

S3 Fig Proportion of total trials rejected per subject according to methodology detailed in S2 Fig. For each subject, all electrodes are considered in aggregate, for a total trial number of 30 x total electrode count for baseline, and 30 x total electrode count x 5 vibration conditions for vibration. The percentage of trials included for analysis is shown in the lighter color, while the percentage of rejected trials is shown in the darker color for both baseline (dark blue) and vibration conditions (teal).
(PDF)

S4 Fig Exemplar subject magnitude-squared coherence (MSC) in the theta (A) and alpha (B) frequency bands for all electrodes. Coherence matrices from Fig 5 are expanded to highlight all coherence changes for individual electrode pairs (lower triangle) as well as region-based averages (upper triangle). Each voxel in the lower triangle represents the average coherence during stimulation (relative to baseline) for two specific electrodes, while the voxels in the upper triangle represent the average coherence for two brain regions. As in Fig 5, the color blocks along the axes correspond to a particular brain region (bottom right of B) and are grouped by left and right hemisphere, and the selected exemplar subject had comparable bilateral electrode coverage across both hemispheres.
(PDF)

S5 Fig Exemplar subject magnitude-squared coherence (MSC) in the theta (A) and alpha (B) frequency bands. Distributions are directly compared between baseline and stimulation conditions. Red asterisks indicate that the global coherence distribution is significantly decreased during stimulation for this exemplar subject, while green asterisks indicate that it is significantly increased during stimulation. Two-tailed Wilcoxon signed-rank test: ** = Bonferroni-corrected *p* < 0.001.
(PDF)

S6 Fig Group-average magnitude-squared coherence (MSC) in the (A) theta and (B) alpha frequency bands for all anatomical regions. MSC plots are shown for 6, 20, and 40 Hz vibration conditions as identified in Fig 3. Because anatomical coverage is clinically driven, there is variation in the number of subjects represented within each coherence voxel. Voxels with an MSC change equal to 0 are pairings that do not exist across any subject. ACC = anterior cingulate cortex; Amyg = amygdala; BG = basal ganglia; Hipp = hippocampus; IFG = inferior frontal gyrus; Occ = occipital lobe; OFC = orbitofrontal cortex; PCC = posterior cingulate cortex; PFC = prefrontal cortex; PHG = parahippocampal gyrus; Temp = temporal lobe; Thal = thalamus.
(PDF)

S7 Fig Theta and alpha coherence changes across all five vibration conditions for OFC and ACC ROIs. The left OFC and ACC were set as seed regions, similar to Fig 8. As in Figs 7 and 8, a threshold of 0.01 was set, and increased coherence during vibration is shown as a positive coherence change, while decreased coherence during vibration is shown as a negative coherence change. Images are shown in radiological view.
(PDF)

S8 Fig Theta and alpha coherence changes across all five vibration conditions for hippocampus and PHG ROIs. The left hippocampus and PHG were set as seed regions, similar to Fig 8. As in Figs 7 and 8, a threshold of 0.01 was set, and increased coherence during vibration is shown as a positive coherence change, while decreased coherence during vibration is shown as a negative coherence change. Images are shown in radiological view.
(PDF)

S9 Fig Baseline coherence across subjects. The average baseline coherence for all four frequency bands is shown for each subject as the mean with error bars representing standard error. Mean baseline coherence was computed by averaging coherence across all electrode pairs. A Kruskal-Wallis test was performed on each frequency band resulting in *p* < 0.001.
(PDF)
