# Supplementary material for: Vibrotactile auricular vagus nerve stimulation alters limbic system connectivity in humans: A pilot study
Source: PLoS One. 2025 May 29;20(5):e0310917. doi: 10.1371/journal.pone.0310917 (PMC12121794; doi:10.1371/journal.pone.0310917)
Supplement: S1 Table — FreeSurfer parcellations were grouped structurally into 28 defined brain regions to reduce the dimensionality. ACC = anterior cingulate cortex; Amyg = amygdala; BG = basal ganglia; Hipp = hippocampus; IFG = inferior frontal gyrus; OFC = orbitofrontal cortex; Occ = occipital lobe; PCC = posterior cingulate cortex; PFC = prefrontal cortex; PHG = parahippocampal gyrus; Temp = temporal lobe; Thal = thalamus. (DOCX) [file pone.0310917.s001.docx]

**S1 Table.** **Aggregated FreeSurfer groups.**

| **Brain Region** | **Corresponding FreeSurfer Parcellations** |
| --- | --- |
| L-ACC | ctx-lh-rostralanteriorcingulate, ctx-lh-caudalanteriorcingulate |
| L-Amyg | Left-Amygdala |
| L-BG | Left-Putamen, Left-Pallidum |
| L-Central | ctx-lh-precentral, ctx-lh-postcentral |
| L-Hipp | Left-Hippocampus |
| L-IFG | ctx-lh-parstriangularis, ctx-lh-parsorbitalis, ctx-lh-parsopercularis |
| L-Insula | ctx-lh-insula |
| L-OFC | ctx-lh-medialorbitofrontal, ctx-lh-lateralorbitofrontal |
| L-Occ | ctx-lh-lingual, ctx-lh-lateraloccipital |
| L-PCC | ctx-lh-posteriorcingulate, ctx-lh-precuneus, ctx-lh-isthmuscingulate |
| L-PFC | ctx-lh-rostralmiddlefrontal, ctx-lh-superiorfrontal |
| L-PHG | ctx-lh-parahippocampal |
| L-Parietal | ctx-lh-supramarginal, ctx-lh-inferiorparietal, ctx-lh-superiorparietal |
| L-Temp | ctx-lh-superiortemporal, ctx-lh-middletemporal, ctx-lh-inferiortemporal, ctx-lh-entorhinal, ctx-lh-fusiform, ctx-lh-temporalpole, ctx-lh-transversetemporal, ctx-lh-bankssts |
| L-Thal | Left-Thalamus |
| R-ACC | ctx-rh-rostralanteriorcingulate |
| R-Amyg | Right-Amygdala |
| R-BG | Right-Putamen |
| R-Central | ctx-rh-precentral, ctx-rh-paracentral |
| R-Hipp | Right-Hippocampus |
| R-IFG | ctx-rh-parstriangularis, ctx-rh-parsorbitalis, ctx-rh-parsopercularis |
| R-Insula | ctx-rh-insula |
| R-OFC | ctx-rh-medialorbitofrontal, ctx-rh-lateralorbitofrontal |
| R-PCC | ctx-rh-posteriorcingulate, ctx-rh-precuneus |
| R-PFC | ctx-rh-rostralmiddlefrontal, ctx-rh-superiorfrontal |
| R-PHG | ctx-rh-parahippocampal |
| R-Parietal | ctx-rh-supramarginal, ctx-rh-inferiorparietal |
| R-Temp | ctx-rh-superiortemporal, ctx-rh-middletemporal, ctx-rh-inferiortemporal, ctx-rh-entorhinal, ctx-rh-fusiform, ctx-rh-temporalpole |

FreeSurfer parcellations were grouped structurally into 28 defined brain regions to reduce the dimensionality. ACC = anterior cingulate cortex; Amyg = amygdala; BG = basal ganglia; Hipp = hippocampus; IFG = inferior frontal gyrus; OFC = orbitofrontal cortex; Occ = occipital lobe; PCC = posterior cingulate cortex; PFC = prefrontal cortex; PHG = parahippocampal gyrus; Temp = temporal lobe; Thal = thalamus
