# Supplementary material for: Vibrotactile auricular vagus nerve stimulation alters limbic system connectivity in humans: A pilot study
Source: PLoS One. 2025 May 29;20(5):e0310917. doi: 10.1371/journal.pone.0310917 (PMC12121794; doi:10.1371/journal.pone.0310917)
Supplement: S2 Table — Bonferroni-corrected post-hoc pairwise tests were run based on statistically significant differences in the Friedman test. Results are reported for all four physiological frequency bands, with significant pairs indicated (corrected p-values). * p < 0.05; ** p < 0.01. (DOCX) [file pone.0310917.s002.docx]

**S2 Table.** **Pairwise comparisons of global coherence changes.**

| **Vibration condition pairs** | | **Corrected p-values and r-values** | | | |
| --- | --- | --- | --- | --- | --- |
| **Vibration 1 (Hz)** | **Vibration 2 (Hz)** | **Theta** | **Alpha** | **Beta** | **Broadband Gamma** |
| 2 | 6 | 1.44e-20**  0.508 | 5.43e-8**  0.310 | 1.33e-8**  0.323 | 5.16e-10**  r = 0.349 |
| 2 | 12 | 3.89e-5**  0.246 | 1.29e-5**  0.258 | 1 | 0.13 |
| 2 | 20 | 2.19e-20**  0.505 | 2.80e-21**  0.517 | 0.014*  0.170 | 9.07e-4**  r = 0.208 |
| 2 | 40 | 3.66e-16**  0.448 | 1.93e-16**  0.452 | 2.94e-7**  0.295 | 0.0071**  r = 0.180 |
| 6 | 12 | 2.91e-7**  0.295 | 1 | 6.41e-6**  0.265 | 4.70e-4**  r = 0.217 |
| 6 | 20 | 1 | 1.28e-5**  0.258 | 0.30 | 0.0086**  r = 0.177 |
| 6 | 40 | 1 | 5.34e-5**  0.242 | 1 | 0.038*  r = 0.154 |
| 12 | 20 | 5.47e-8**  0.310 | 2.66e-8**  0.317 | 0.47 | 1 |
| 12 | 40 | 1.06e-4**  0.234 | 4.00e-7**  0.292 | 4.72e-6**  0.268 | 1 |
| 20 | 40 | 1 | 1 | 0.15 | 1 |

Bonferroni-corrected post-hoc pairwise tests were run based on statistically significant differences in the Friedman test. Results are reported for all four physiological frequency bands, with significant pairs indicated (corrected *p*-values). Effect sizes for significant pairs are listed below. * *p* < 0.05; ** *p* < 0.01.
