# Supplementary material for: Vibrotactile auricular vagus nerve stimulation alters limbic system connectivity in humans: A pilot study
Source: PLoS One. 2025 May 29;20(5):e0310917. doi: 10.1371/journal.pone.0310917 (PMC12121794; doi:10.1371/journal.pone.0310917)
Supplement: S3 Table — For each of five ROIs, brain regions with a coherence change of at least 0.01 (absolute value) are listed. Response regions are categorized by both vibration frequency (2, 6, 12, 20, and 40 Hz) and coherence frequency (theta and alpha). The ROIs were set as the seed and the coherence was computed with all other brain regions, and then the response threshold was applied. The regions listed here correspond to the regions depicted visually in Fig 8 and S7 and S8 Figs. Note that the regions listed here are based on the response threshold rather than a statistical test. ACC = anterior cingulate cortex; Amyg = amygdala; BG = basal ganglia; Hipp = hippocampus; IFG = inferior frontal gyrus; OFC = orbitofrontal cortex; Occ = occipital lobe; PCC = posterior cingulate cortex; PFC = prefrontal cortex; PHG = parahippocampal gyrus; Temp = temporal lobe; Thal = thalamus. (DOCX) [file pone.0310917.s003.docx]

**S3 Table.** **Threshold-applied responses to ROIs.**

| **Seed** | **Vibration Frequency (Hz)** | **Threshold-applied responder regions** | |
| --- | --- | --- | --- |
|  |  | **Theta coherence** | **Alpha coherence** |
| L-OFC | 2 | Decrease: L-Central, R-IFG, R-PHG, R-Parietal | Increase: R-PCC  Decrease: L-Central, R-Amyg, R-Hipp, R-Insula, R-PFC |
|  | 6 | Increase: L-ACC, L-Central, L-Hipp, L-IFG, L-Insula, L-PCC, L-PFC, L-Parietal, L-Temp, L-Thal, R-Amyg, R-Hipp, R-Insula, R-OFC, R-PFC, R-Temp | Increase: L-Occ, R-PHG |
|  | 12 | Increase: L-IFG, L-Thal, R-Insula  Decrease: R-PHG, R-Parietal | Decrease: L-Amyg, R-PFC |
|  | 20 | Increase: L-ACC, L-BG, L-Hipp, L-IFG, L-Insula, L-Occ, L-PFC, L-Parietal, L-Temp, R-Temp | Increase: L-BG, L-Insula, L-Occ, L-PFC, L-Parietal, L-Thal |
|  | 40 | Increase: L-ACC, L-Hipp, L-IFG, L-PFC, L-Temp, L-Thal, R-Amyg, R-Insula, R-OFC, R-PHG, R-Parietal | Increase: L-Parietal, L-Thal, R-ACC |
| L-ACC | 2 | Increase: L-BG, R-PHG  Decrease: L-Hipp, L-Occ, L-PHG, L-Temp, R-ACC, R-Hipp, R-IFG, R-Insula, R-OFC, R-Temp | Increase: R-PHG  Decrease: L-PHG, L-Temp, R-ACC, R-Amyg, R-Central, R-OFC, R-PFC, R-Temp |
|  | 6 | Increase: L-BG, L-IFG, L-OFC, L-PFC, R-PFC, R-PHG  Decrease: L-Occ, R-Central | Increase: L-Amyg, L-BG, R-Hipp, R-PHG  Decrease: L-Occ, L-PHG, R-Temp |
|  | 12 | Increase: L-Amyg, L-BG, R-Amyg, R-Insula, R-PHG  Decrease: L-Occ, L-PHG, R-Central | Increase: L-Amyg, L-BG, R-PHG  Decrease: L-PHG, R-PFC |
|  | 20 | Increase: L-BG, L-IFG, L-Insula, L-OFC, L-PCC, L-PFC, R-ACC, R-PHG  Decrease: R-Hipp, R-OFC | Increase: L-Amyg, L-BG, L-Occ, R-ACC, R-Central, R-PCC, R-PHG  Decrease: R-OFC |
|  | 40 | Increase: L-BG, L-IFG, L-Insula, L-OFC, L-PFC, R-IFG, R-PFC, R-PHG  Decrease: L-Hipp, L-Occ, L-PCC, R-Central | Increase: L-Amyg, L-BG, L-PFC, R-ACC, R-PHG, R-Parietal  Decrease: L-Occ, L-PCC |
| L-Amyg | 2 | Increase: L-Central, L-IFG, L-PCC, R-BG  Decrease: L-Parietal, R-ACC | Increase: L-Occ, R-BG, R-Central, R-PCC  Decrease: L-Thal, R-ACC, R-OFC, R-PFC |
|  | 6 | Increase: L-Central, L-Thal, R-BG, R-Central, R-PHG  Decrease: L-Parietal, R-ACC, R-Parietal | Increase: L-ACC, R-BG, R-Central, R-Hipp, R-PCC, R-PHG |
|  | 12 | Increase: L-ACC, L-Central, R-Hipp, R-PFC  Decrease: L-Occ, L-PHG, R-ACC, R-PHG, R-Parietal | Increase: L-ACC, L-Parietal, R-BG, R-IFG, R-PCC, R-PHG  Decrease: L-OFC, R-ACC |
|  | 20 | Increase: L-BG, R-BG, R-Central  Decrease: L-Occ | Increase: L-ACC, L-Parietal, R-BG, R-Central, R-IFG, R-PCC, R-PHG |
|  | 40 | Increase: L-BG, L-Hipp, L-Insula, R-ACC, R-Amyg, R-Central, R-PCC, R-Parietal  Decrease: R-BG | Increase: L-ACC, L-Hipp, L-Insula, L-Parietal, R-ACC, R-BG, R-PCC, R-PHG, R-Parietal  Decrease: L-Thal |
| L-Hipp | 2 | Increase: L-BG, L-Thal  Decrease: L-ACC, L-Occ, R-ACC, R-IFG, R-OFC, R-PFC, R-PHG | Increase: R-BG, R-PCC  Decrease: L-Central, R-Amyg, R-Insula, R-OFC, R-PFC |
|  | 6 | Increase: L-OFC, R-Central  Decrease: L-Occ, R-ACC, R-OFC | Increase: R-BG, R-PCC, R-PHG  Decrease: R-Central |
|  | 12 | Increase: R-BG, R-Central  Decrease: L-Insula, L-Occ, L-PFC, R-OFC, R-PCC, R-PHG, R-Parietal | Increase: R-BG, R-PCC  Decrease: L-Occ, R-ACC, R-Amyg, R-Central, R-OFC, R-PFC |
|  | 20 | Increase: L-BG, L-OFC  Decrease: L-Occ, L-PHG, R-BG, R-IFG, R-OFC | Increase: R-BG, R-PHG  Decrease: L-Central, L-Occ, R-Amyg |
|  | 40 | Increase: L-Amyg, L-BG, L-OFC, R-PCC, R-PHG  Decrease: L-ACC, L-Occ | Increase: L-Amyg, R-BG, R-PCC, R-PHG |
| L-PHG | 2 | Increase: R-Amyg, R-PCC  Decrease: L-ACC, L-Occ, R-ACC, R-IFG, R-PFC | Increase: L-Thal  Decrease: L-ACC, L-Occ |
|  | 6 | Decrease: L-Occ, R-Central, R-IFG, R-Insula, R-OFC, R-PFC, R-PHG, R-Parietal | Increase: L-Occ, L-Thal  Decrease: L-ACC, L-IFG, R-ACC, R-OFC |
|  | 12 | Increase: L-Central  Decrease: L-ACC, L-Amyg, L-Occ, R-ACC, R-Central, R-IFG, R-PFC, R-PHG, R-Parietal | Increase: L-Thal, R-Hipp, R-Insula  Decrease: L-ACC, R-ACC, R-Central, R-PHG |
|  | 20 | Increase: R-Central, R-Hipp, R-PCC, R-PHG  Decrease: L-Hipp, L-Insula, L-Occ, R-IFG, R-PFC | Increase: L-Thal, R-Central, R-Insula, R-PCC  Decrease: R-OFC, R-PHG, R-Parietal |
|  | 40 | Increase: R-Hipp, R-PCC, R-PHG  Decrease: L-Occ, L-Thal | Increase: L-Thal, R-Hipp, R-OFC, R-PCC, R-PFC, R-PHG |

For each of five ROIs, brain regions with a coherence change of at least 0.01 (absolute value) are listed. Response regions are categorized by both vibration frequency (2, 6, 12, 20, and 40 Hz) and coherence frequency (theta and alpha). The ROIs were set as the seed and the coherence was computed with all other brain regions before applying the response threshold. The regions listed here correspond to the regions depicted visually in Fig 8 and S7 and S8 Figs. Note that the regions listed here are based on the response threshold rather than a statistical test. ACC = anterior cingulate cortex; Amyg = amygdala; BG = basal ganglia; Hipp = hippocampus; IFG = inferior frontal gyrus; OFC = orbitofrontal cortex; Occ = occipital lobe; PCC = posterior cingulate cortex; PFC = prefrontal cortex; PHG = parahippocampal gyrus; Temp = temporal lobe; Thal = thalamus
