# Supplementary material for: Vibrotactile auricular vagus nerve stimulation alters limbic system connectivity in humans: A pilot study
Source: PLoS One. 2025 May 29;20(5):e0310917. doi: 10.1371/journal.pone.0310917 (PMC12121794; doi:10.1371/journal.pone.0310917)
Supplement: S4 Table — Kruskal-Wallis tests were run to compare the threshold-applied response distributions of theta and alpha coherence changes to each of five ROIs. The ROIs were set as seeds and coherence values to all other brain regions were computed. A response threshold was set at 0.01 (absolute value). All seeds are in the left hemisphere. * p < 0.05; ** p < 0.01. (DOCX) [file pone.0310917.s004.docx]

**S4 Table.** **Comparisons of group-level coherence responses to ROIs.**

| **Seed** | **p-values (Kruskal-Wallis)** | |
| --- | --- | --- |
|  | **Theta** | **Alpha** |
| Orbitofrontal cortex | 0.024* | 0.039* |
| Anterior cingulate cortex | 0.033* | 0.001** |
| Amygdala | 0.201 | 0.508 |
| Hippocampus | 0.529 | 0.403 |
| Parahippocampal gyrus | 0.146 | 0.286 |

Kruskal-Wallis tests were run to compare the threshold-applied response distributions of theta and alpha coherence changes to each of five ROIs. The ROIs were set as seeds and coherence values to all other brain regions were computed. A response threshold was set at 0.01 (absolute value). All seeds are in the left hemisphere. * *p* < 0.05; ** *p* < 0.01.
