# Supplementary material for: Vibrotactile auricular vagus nerve stimulation alters limbic system connectivity in humans: A pilot study
Source: PLoS One. 2025 May 29;20(5):e0310917. doi: 10.1371/journal.pone.0310917 (PMC12121794; doi:10.1371/journal.pone.0310917)
Supplement: S5 Table — Bonferroni-corrected post-hoc pairwise tests were run based on statistically significant differences in the Kruskal-Wallis test (S4 Table). Results are reported for both theta and alpha coherence distributions, with significant pairs indicated (corrected p-values). * p < 0.05; ** p < 0.01. (DOCX) [file pone.0310917.s005.docx]

**S5 Table.** **Pairwise comparisons of group-level coherence responses to significant ROIs.**

| **Seed** | **Vibration pairs** | | **Corrected p-values** | |
| --- | --- | --- | --- | --- |
|  | **Vibration 1 Frequency** | **Vibration 2 Frequency** | **Theta** | **Alpha** |
| Orbitofrontal cortex | 2 | 6 | 0.125 | 0.223 |
|  | 2 | 12 | 0.456 | 1 |
|  | 2 | 20 | 0.014* | 0.159 |
|  | 2 | 40 | 0.033* | 1 |
|  | 6 | 12 | 1 | 0.506 |
|  | 6 | 20 | 1 | 1 |
|  | 6 | 40 | 1 | 1 |
|  | 12 | 20 | 1 | 0.697 |
|  | 12 | 40 | 1 | 1 |
|  | 20 | 40 | 1 | 1 |
| Anterior cingulate cortex | 2 | 6 | 0.068 | 0.021* |
|  | 2 | 12 | 0.713 | 0.310 |
|  | 2 | 20 | 0.069 | 0.001** |
|  | 2 | 40 | 0.311 | 0.128 |
|  | 6 | 12 | 1 | 1 |
|  | 6 | 20 | 1 | 1 |
|  | 6 | 40 | 1 | 1 |
|  | 12 | 20 | 1 | 1 |
|  | 12 | 40 | 1 | 1 |
|  | 20 | 40 | 1 | 1 |

Bonferroni-corrected post-hoc pairwise tests were run based on statistically significant differences in the Kruskal-Wallis test (S4 Table). Results are reported for both theta and alpha coherence distributions, with significant pairs indicated (corrected *p*-values). * *p* < 0.05; ** *p* < 0.01.
