# Supplementary material for: Vibrotactile auricular vagus nerve stimulation alters limbic system connectivity in humans: A pilot study
Source: PLoS One. 2025 May 29;20(5):e0310917. doi: 10.1371/journal.pone.0310917 (PMC12121794; doi:10.1371/journal.pone.0310917)
Supplement: S6 Table — Bonferroni-corrected post-hoc pairwise tests were run based on statistically significant differences in the Kruskal-Wallis test. Results are reported for all four physiological frequency bands, with significant pairs indicated (corrected p-values). * p < 0.05; ** p < 0.01. (DOCX) [file pone.0310917.s006.docx]

**S6 Table.** **Pairwise comparisons of baseline coherence.**

| **Subject pairs** | | **Corrected p-values** | | | |
| --- | --- | --- | --- | --- | --- |
| **Subject 1 ID** | **Subject 2 ID** | **Theta** | **Alpha** | **Beta** | **Broadband Gamma** |
| 1 | 2 | 1 | 0.096 | 1.47e-15** | 1.75e-18** |
| 1 | 3 | 1 | 1 | 0.0019** | 1.74e-7** |
| 1 | 4 | 0.0045** | 3.26e-5** | 1 | 5.15e-12** |
| 1 | 5 | 6.26e-5** | 1.09e-4** | 3.70e-9** | 0.80 |
| 1 | 6 | 0.012* | 1 | 0.0036** | 1.97e-15** |
| 1 | 7 | 1 | 1 | 0.27 | 1 |
| 2 | 3 | 0.032* | 0.28 | 0.077 | 1 |
| 2 | 4 | 1.07e-7** | 2.47e-14** | 1.97e-12** | 1 |
| 2 | 5 | 0.0099** | 0.44 | 1 | 2.16e-11** |
| 2 | 6 | 0.90 | 0.18 | 8.73e-4** | 1 |
| 2 | 7 | 1 | 0.27 | 1.18e-7** | 4.83e-27** |
| 3 | 4 | 0.94 | 2.94e-4** | 0.0088** | 1 |
| 3 | 5 | 4.09e-7** | 0.0010** | 1 | 9.60e-4** |
| 3 | 6 | 1.84e-4** | 1 | 1 | 1 |
| 3 | 7 | 1 | 1 | 1 | 2.69e-11** |
| 4 | 5 | 6.30e-15** | 7.51e-19** | 1.93e-7** | 4.31e-7** |
| 4 | 6 | 1.27e-10** | 1.17e-5** | 0.019* | 1 |
| 4 | 7 | 9.87e-4** | 2.18e-6** | 0.72 | 3.67e-17** |
| 5 | 6 | 1 | 2.41e-4** | 0.24 | 1.51e-9** |
| 5 | 7 | 1.14e-4** | 3.62e-4** | 0.0012** | 0.0089** |
| 6 | 7 | 0.029* | 1 | 1 | 6.69e-22** |

Bonferroni-corrected post-hoc pairwise tests were run based on statistically significant differences in the Kruskal-Wallis test. Results are reported for all four physiological frequency bands, with significant pairs indicated (corrected *p*-values). * *p* < 0.05; ** *p* < 0.01.
