# Supplementary material for: Vibrotactile auricular vagus nerve stimulation alters limbic system connectivity in humans: A pilot study
Source: PLoS One. 2025 May 29;20(5):e0310917. doi: 10.1371/journal.pone.0310917 (PMC12121794; doi:10.1371/journal.pone.0310917)
Supplement: S1 Fig — ACC = anterior cingulate cortex; Amyg = amygdala; BG = basal ganglia; Hipp = hippocampus; IFG = inferior frontal gyrus; Occ = occipital lobe; OFC = orbitofrontal cortex; PCC = posterior cingulate cortex; PFC = prefrontal cortex; PHG = parahippocampal gyrus; Temp = temporal lobe; Thal = thalamus. (PDF) [file pone.0310917.s007.pdf]

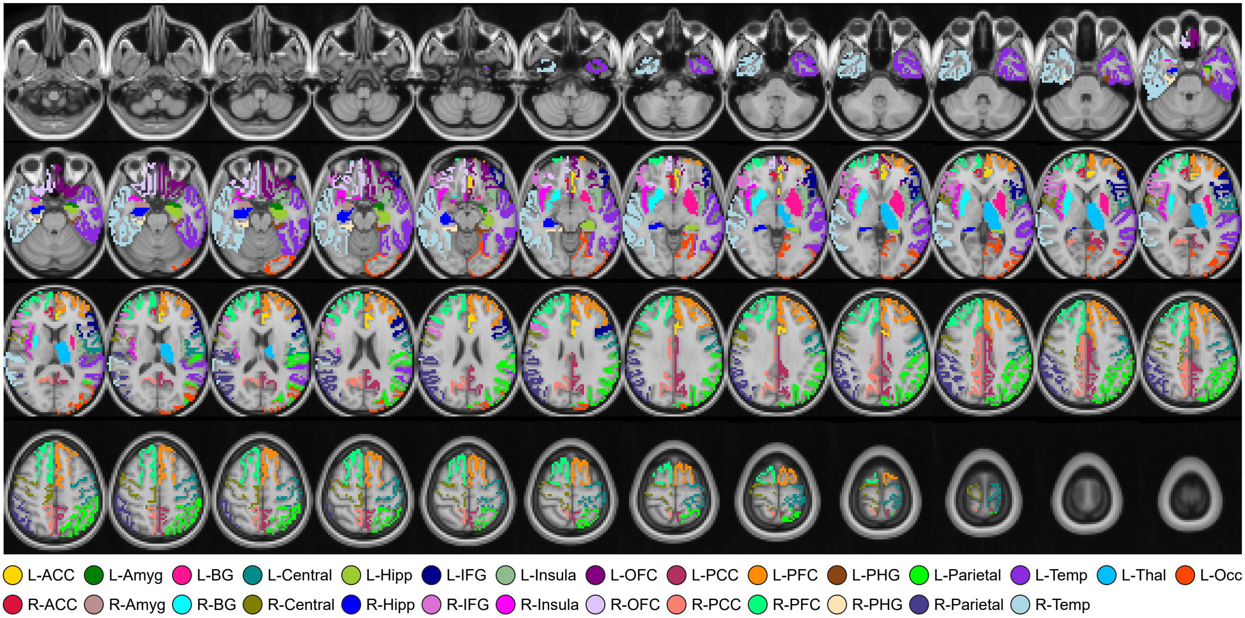
L-ACC
L-Amyg
L-BG
L-Central
L-Hipp
L-IFG
L-Insula
L-OFC
L-PCC
L-PFC
L-PHG
L-Parietal
L-Temp
L-Thal
L-Occ
R-ACC
R-Amyg
R-BG
R-Central
R-Hipp
R-IFG
R-Insula
R-OFC
R-PCC
R-PFC
R-PHG
R-Parietal
R-Temp
