# Supplementary figures and images for: Vibrotactile auricular vagus nerve stimulation alters limbic system connectivity in humans: A pilot study
Source: PLoS One. 2025 May 29;20(5):e0310917. doi: 10.1371/journal.pone.0310917 (PMC12121794; doi:10.1371/journal.pone.0310917)

## A. Preprocessed signals

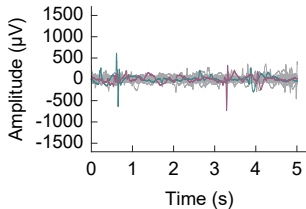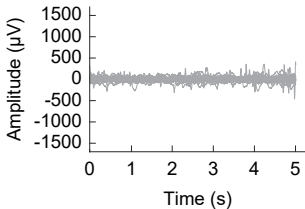

## B. Electrode location

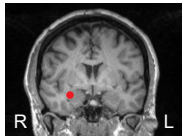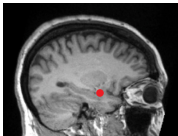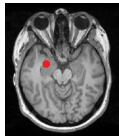

Supplement: S2 Fig — (A) All preprocessed baseline epochs (5 s) from one electrode in one subject. Any trial with a positive or negative spike exceeding abs(500) µV (two trials highlighted here in teal and pink) was marked as containing epileptiform discharges and excluded from future analysis. (B) This example electrode is located in the right amygdala. (PDF) [file pone.0310917.s008.pdf]

# Rejected Trials Across all Electrodes

Baseline (keep/reject)  
Vibration (keep/reject)

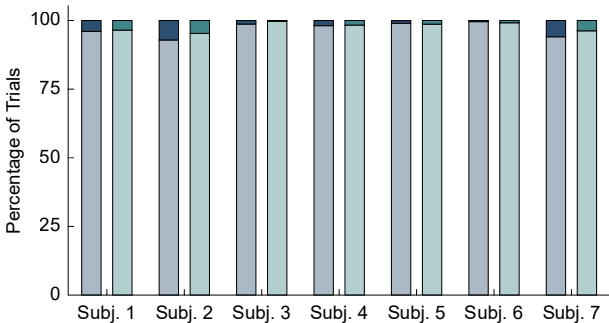

Supplement: S3 Fig — For each subject, all electrodes are considered in aggregate, for a total trial number of 30 x total electrode count for baseline, and 30 x total electrode count x 5 vibration conditions for vibration. The percentage of trials included for analysis is shown in the lighter color, while the percentage of rejected trials is shown in the darker color for both baseline (dark blue) and vibration conditions (teal). (PDF) [file pone.0310917.s009.pdf]

# A. Theta coherence

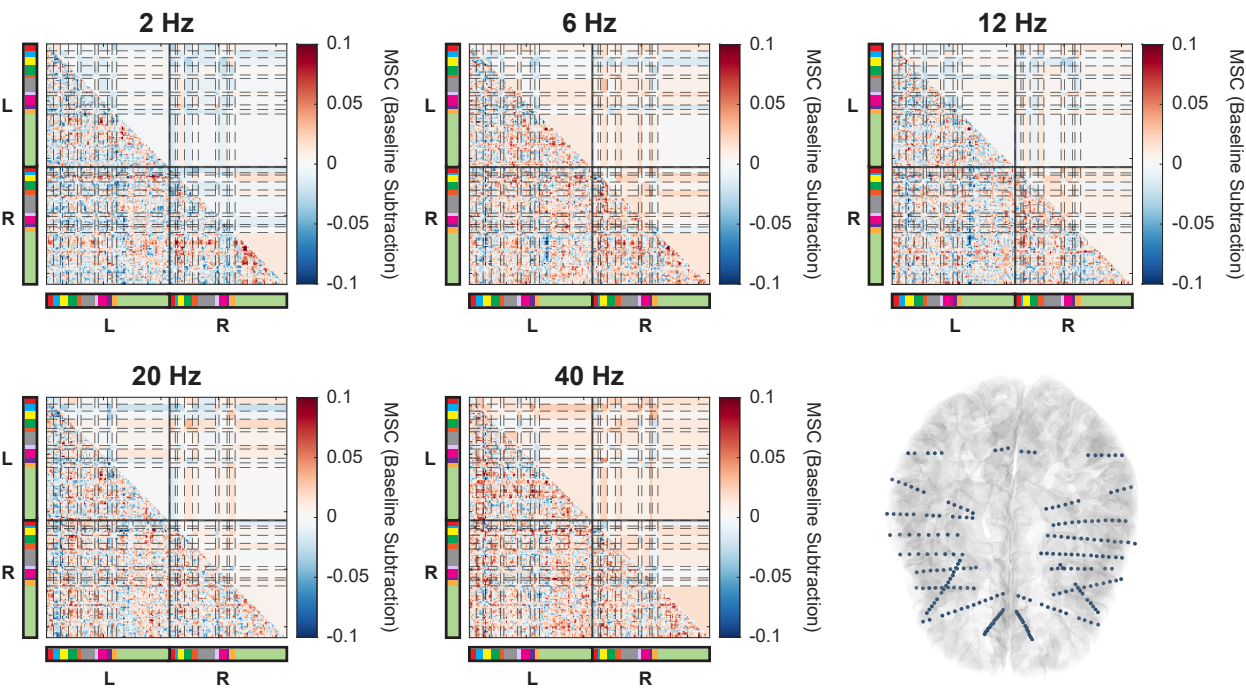

# B. Alpha coherence

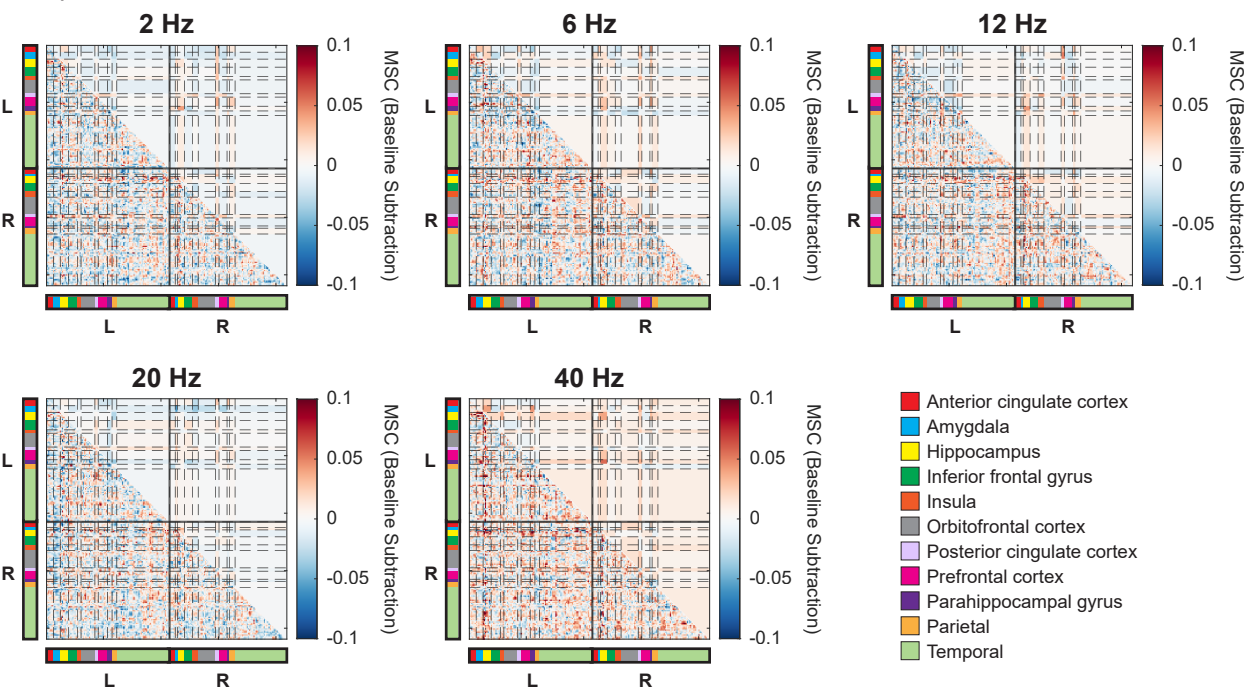

Supplement: S4 Fig — Coherence matrices from Fig 5 are expanded to highlight all coherence changes for individual electrode pairs (lower triangle) as well as region-based averages (upper triangle). Each voxel in the lower triangle represents the average coherence during stimulation (relative to baseline) for two specific electrodes, while the voxels in the upper triangle represent the average coherence for two brain regions. As in Fig 5, the color blocks along the axes correspond to a particular brain region (bottom right of B) and are grouped by left and right hemisphere, and the selected exemplar subject had comparable bilateral electrode coverage across both hemispheres. (PDF) [file pone.0310917.s010.pdf]

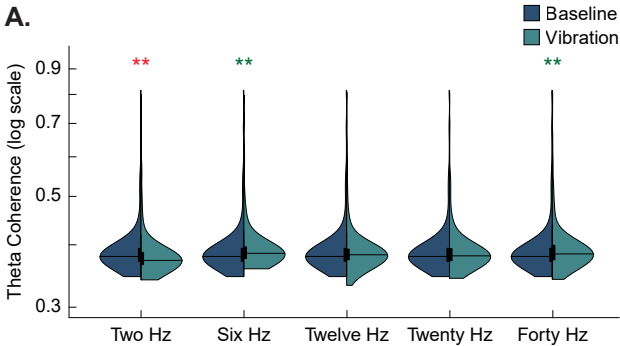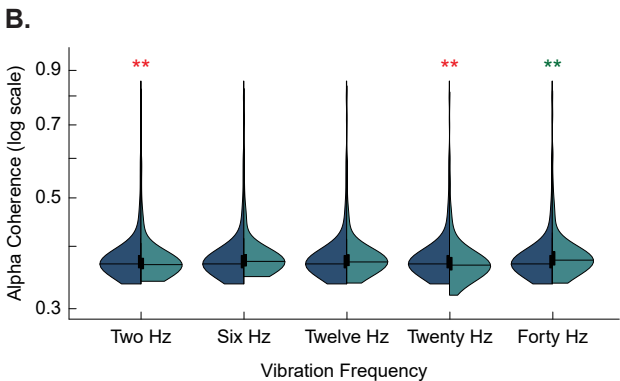

Supplement: S5 Fig — Distributions are directly compared between baseline and stimulation conditions. Red asterisks indicate that the global coherence distribution is significantly decreased during stimulation for this exemplar subject, while green asterisks indicate that it is significantly increased during stimulation. Two-tailed Wilcoxon signed-rank test: ** = Bonferroni-corrected p < 0.001. (PDF) [file pone.0310917.s011.pdf]

## A. Theta coherence

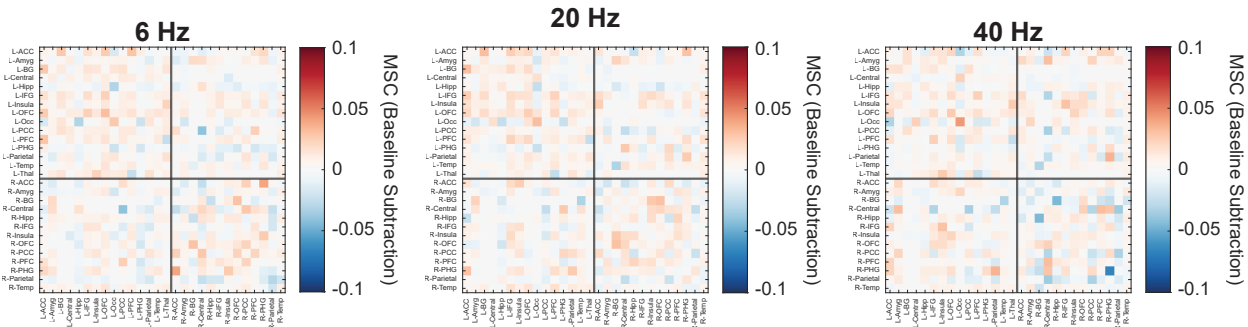

## B. Alpha coherence

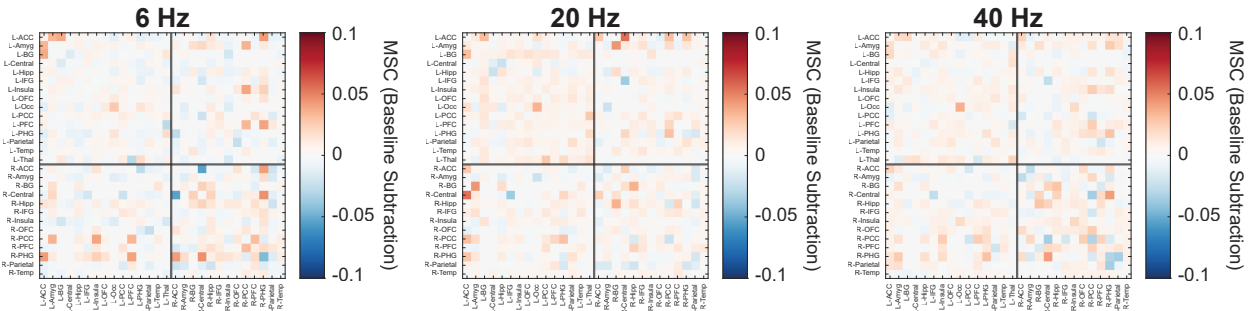

Supplement: S6 Fig — MSC plots are shown for 6, 20, and 40 Hz vibration conditions as identified in Fig 3. Because anatomical coverage is clinically driven, there is variation in the number of subjects represented within each coherence voxel. Voxels with an MSC change equal to 0 are pairings that do not exist across any subject. ACC = anterior cingulate cortex; Amyg = amygdala; BG = basal ganglia; Hipp = hippocampus; IFG = inferior frontal gyrus; Occ = occipital lobe; OFC = orbitofrontal cortex; PCC = posterior cingulate cortex; PFC = prefrontal cortex; PHG = parahippocampal gyrus; Temp = temporal lobe; Thal = thalamus. (PDF) [file pone.0310917.s012.pdf]

**A.**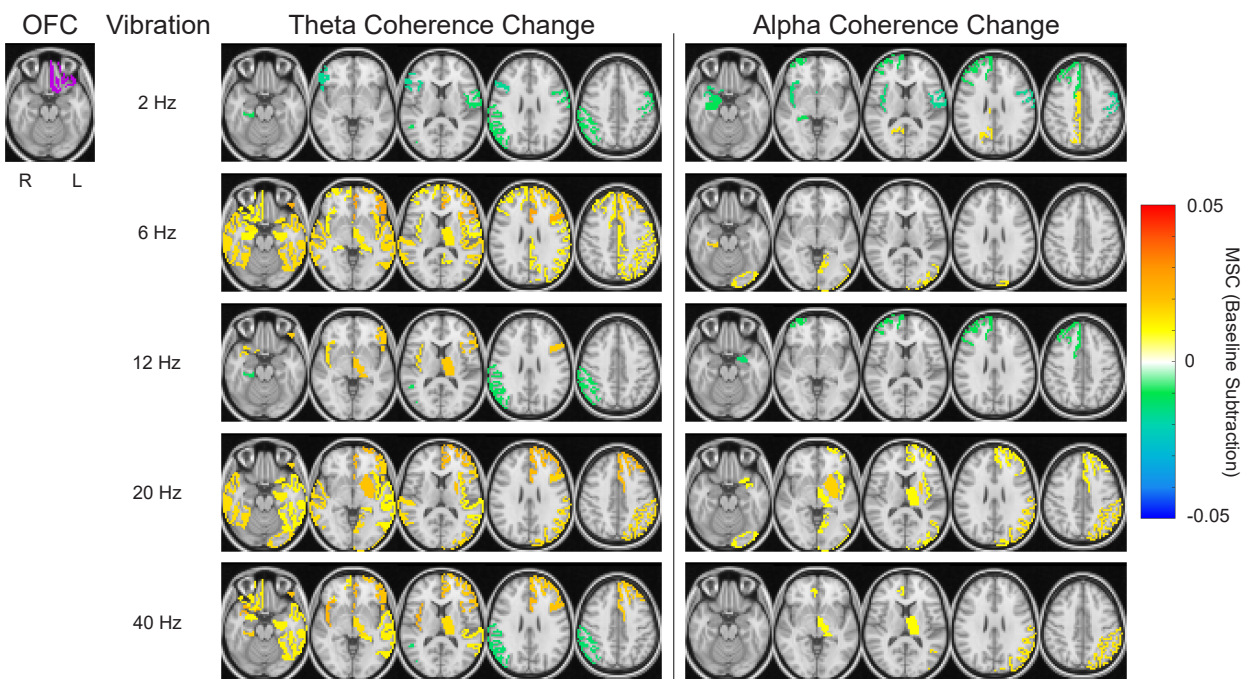**B.**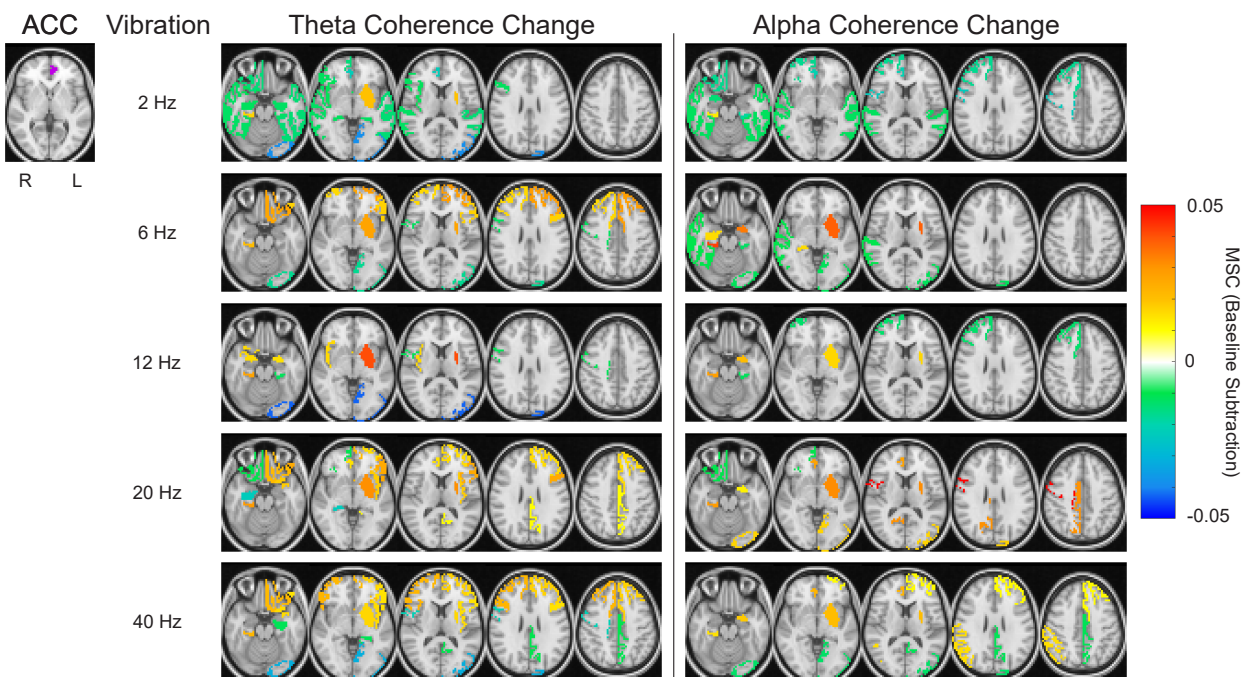

Supplement: S7 Fig — The left OFC and ACC were set as seed regions, similar to Fig 8. As in Figs 7 and 8, a threshold of 0.01 was set, and increased coherence during vibration is shown as a positive coherence change, while decreased coherence during vibration is shown as a negative coherence change. Images are shown in radiological view. (PDF) [file pone.0310917.s013.pdf]

**A.**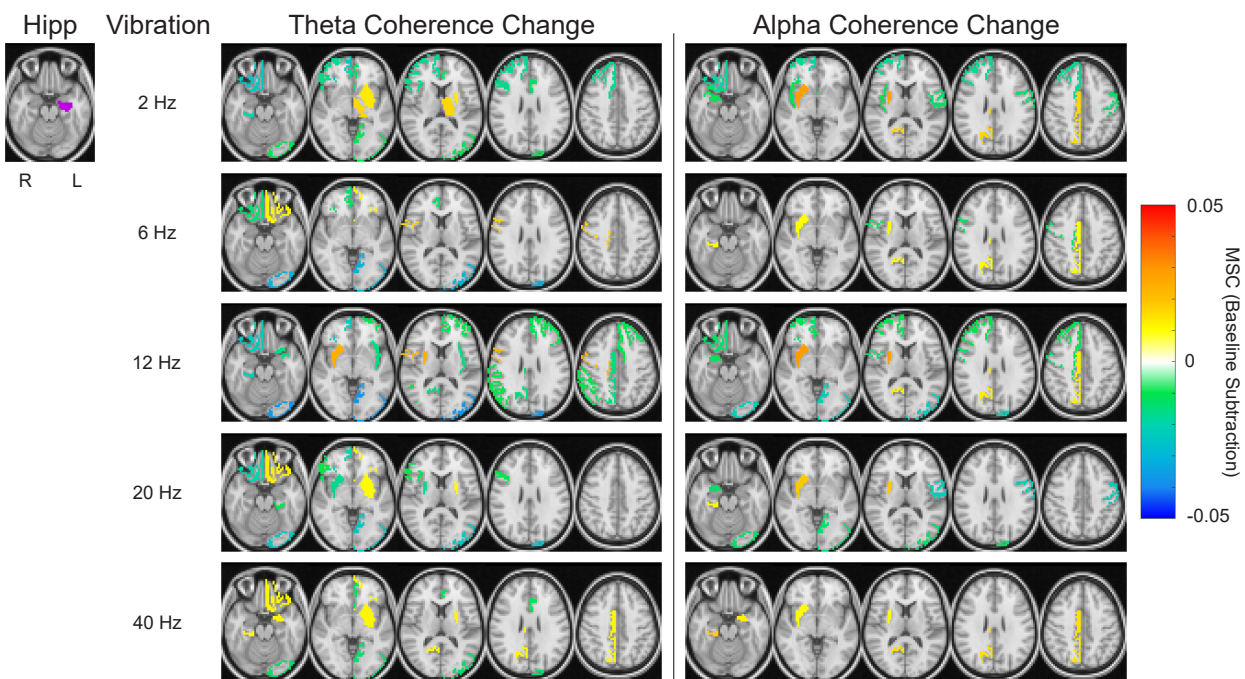**B.**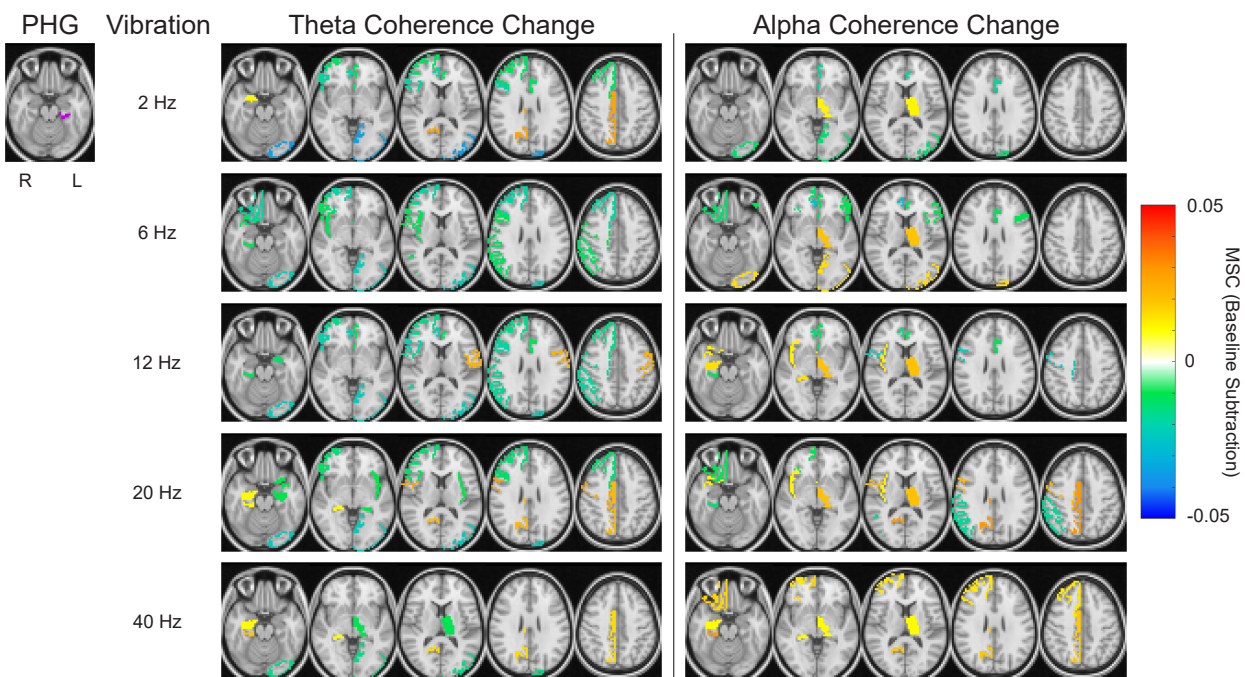

Supplement: S8 Fig — The left hippocampus and PHG were set as seed regions, similar to Fig 8. As in Figs 7 and 8, a threshold of 0.01 was set, and increased coherence during vibration is shown as a positive coherence change, while decreased coherence during vibration is shown as a negative coherence change. Images are shown in radiological view. (PDF) [file pone.0310917.s014.pdf]

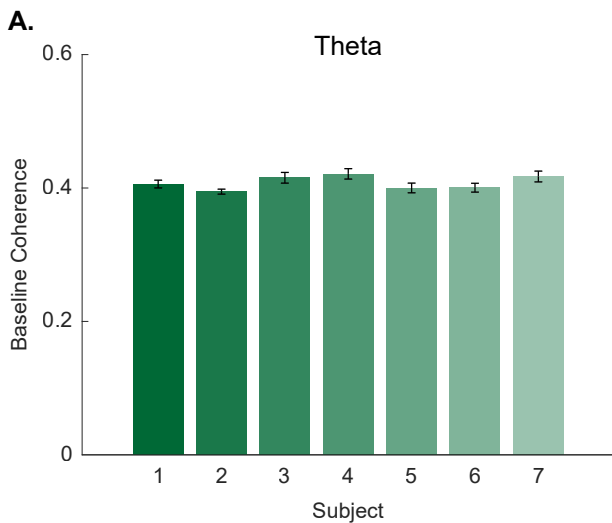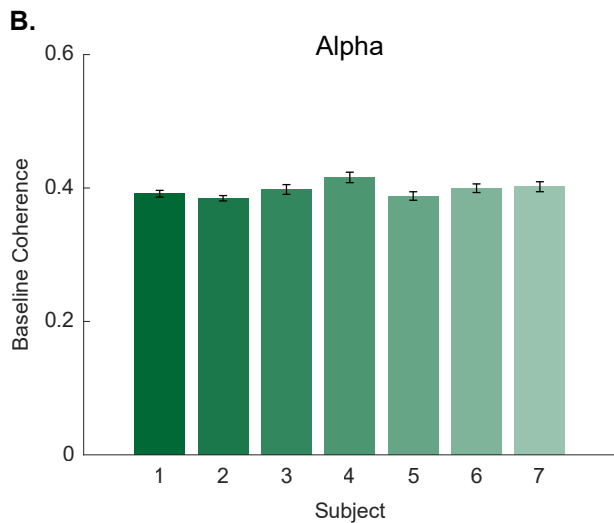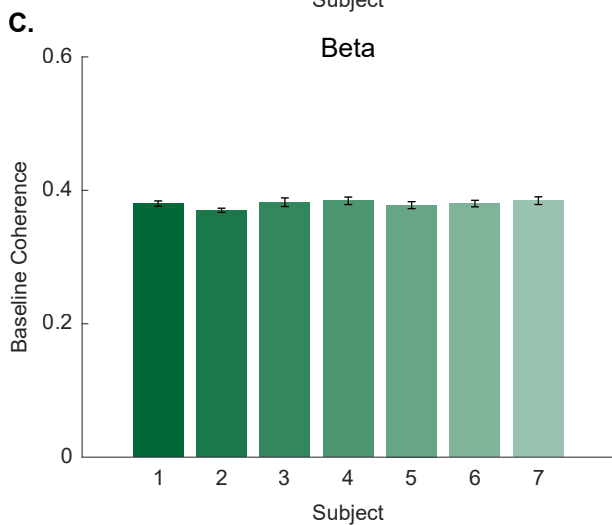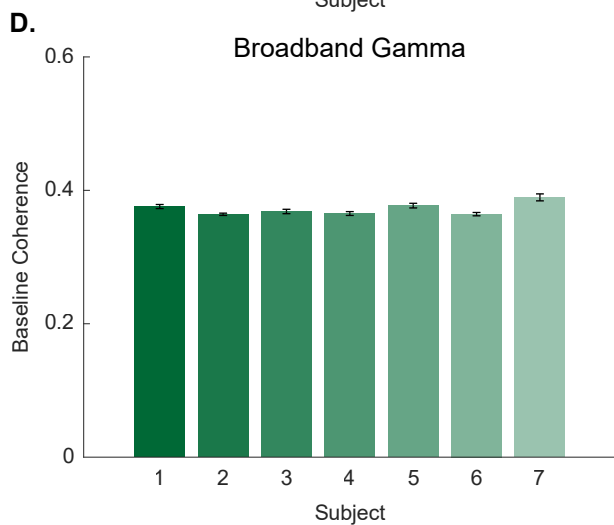

Supplement: S9 Fig — The average baseline coherence for all four frequency bands is shown for each subject as the mean with error bars representing standard error. Mean baseline coherence was computed by averaging coherence across all electrode pairs. A Kruskal-Wallis test was performed on each frequency band resulting in p < 0.001. (PDF) [file pone.0310917.s015.pdf]
